# Identifying yield-related genes in maize based on ear trait plasticity

**DOI:** 10.1186/s13059-023-02937-6

**Published:** 2023-04-25

**Authors:** Minguo Liu, Shuaisong Zhang, Wei Li, Xiaoming Zhao, Xi-Qing Wang

**Affiliations:** 1 State Key Laboratory of Plant Environmental Resilience, College of Biological Sciences, China Agricultural University, Beijing, 100193 China; 2 Frontier Technology Research Institute of China Agricultural University in Shenzhen, Shenzhen, 518000 China; 3 Center for Crop Functional Genomics and Molecular Breeding, China Agricultural University, Beijing, 100193 China

**Keywords:** Maize, Ear phenotypes, Phenotypic plasticity, Transgenic maize inbred population, Automated ear phenotyping platform, MAIZTRO

## Abstract

**Background:**

Phenotypic plasticity is defined as the phenotypic variation of a trait when an organism is exposed to different environments, and it is closely related to genotype. Exploring the genetic basis behind the phenotypic plasticity of ear traits in maize is critical to achieve climate-stable yields, particularly given the unpredictable effects of climate change. Performing genetic field studies in maize requires development of a fast, reliable, and automated system for phenotyping large numbers of samples.

**Results:**

Here, we develop MAIZTRO as an automated maize ear phenotyping platform for high-throughput measurements in the field. Using this platform, we analyze 15 common ear phenotypes and their phenotypic plasticity variation in 3819 transgenic maize inbred lines targeting 717 genes, along with the wild type lines of the same genetic background, in multiple field environments in two consecutive years. Kernel number is chosen as the primary target phenotype because it is a key trait for improving the grain yield and ensuring yield stability. We analyze the phenotypic plasticity of the transgenic lines in different environments and identify 34 candidate genes that may regulate the phenotypic plasticity of kernel number.

**Conclusions:**

Our results suggest that as an integrated and efficient phenotyping platform for measuring maize ear traits, MAIZTRO can help to explore new traits that are important for improving and stabilizing the yield. This study indicates that genes and alleles related with ear trait plasticity can be identified using transgenic maize inbred populations.

**Supplementary Information:**

The online version contains supplementary material available at 10.1186/s13059-023-02937-6.

## Background

Grain production is critical to food security and depends on many factors attributed to gene-environment interactions [1, 2]. Climate change has become an indisputable fact, with inevitable impacts on the earth including rising temperatures, alarmingly high atmospheric levels of carbon dioxide and other greenhouse gasses, unpredictable rainfall, and increasingly common extreme weather conditions, all of which pose significant direct or indirect threats to crop growth and grain production [3, 4]. Therefore, exploring the regulation of gene-environment interactions and improving yield stability of grain crops in diverse and uncertain environments is of critical importance in the face of increasing food demand and climate change.

Plants have evolved unique mechanisms for responding to environmental changes, as they are fixed in location and cannot seek shelter or change their environment [5, 6]. This is reflected in the phenotypic plasticity of plants, which describes the phenotypic variation of a trait in different environments; this plastic response to the environment is referred to as gene-environment interaction and is an important factor in plant breeding [7–9]. Phenotypic plasticity is heritable and is under selection during breeding [10, 11]. The phenotype that arises in any particular environment can be dissected and predicted by manipulating the relative contribution of genetic components and phenotypic plasticity [11]. Some phenotypic plasticity can contribute to heterosis through hybridization [11]. Although some studies have confirmed that there is a close relationship between phenotypic plasticity and genetic structure, there is currently no clear understanding of the regulatory genes involved [12, 13]. Identification of these genes would facilitate breeding of optimized varieties that can take advantage of certain environmental conditions or are more widely adaptable to environmental changes, and transgene technology provides a powerful means for achieving this goal [7, 14]. Conventional transgene technology focuses on a limited number of genes and controlled environments, however, and thus there is often a gap between gene performance in a controlled environment versus the field [15, 16]. The method-based performance assessment of transgenic populations with high gene diversity in the field may offer a fresh viewpoint for screening genes involved in yield stability and aid in the application of transgene technology to mitigate the unfavorable effects of a rapidly changing climate on grain production.

Maize (*Zea mays*) is an important staple grain crop widely used for human and livestock consumption, biofuels, and industrial feedstock [7, 11]. It is planted in different ecological, climatic, and geographical conditions, all of which affect its yield [10]. Therefore, exploring maize yield stability in a variety of environments is of great significance to achieving stable grain production [11, 17]. Unfortunately, the extreme complexity and low heritability of yield as a quantitative trait lead to low breeding efficiency and a poor understanding of the genetic structure affecting yield [18, 19]. Some yield-related traits have higher heritability and better stability under different growth conditions [20] and have been used to improve maize breeding in many studies [21]. An important focus in maize yield studies is exploring the phenotypic plasticity of maize ears, with a particular focus on kernel number per ear—a key trait in high-yield maize breeding [22].

Efficient and accurate characterization of ear phenotypes in different environments is fundamental to plasticity research. Although modern harvesting equipment can automatically and efficiently measure traits such as ear number, bulk density, and kernel water content in the field, the characterization of other traits such as kernel number per ear, which are significantly regulated by genetics, must be done manually [22–25]. Manual measurement is inefficient, subjective, prone to error, and able to generate only limited indices. Although some previous studies have tried to explore the effectiveness of automated measurement methods, they are limited by efficiency or lack of several key ear phenotypes. One such method treats images on a homogenous background with traditional image processing technology, capturing each kernel as an object and obtaining the length and width of the kernel [26]. It can be difficult to use this method to obtain maize kernel objects on an ear, however, due the nonhomologous background (i.e., other kernels on the ear). Miller et al. [27] have solved this problem; however, they ignored key ear traits such as the number of kernels per ear and the number of rows per ear. Warman et al. [28] proposed novel algorithms to focus on these ear traits, but the approach is limited by the simplicity of the imaging platform (a non-product concept device) and low analysis efficiency (one ear per analysis). Therefore, there is a need to develop a high-throughput automated phenotyping platform that integrates the ear phenotypes concerned by Miller et al. [27] and Warman et al. [28], as well as many other critical phenotypic characteristics.

To this end, we developed a maize ear phenotyping platform called MAIZTRO that combines all necessary hardware and software and allows efficient, accurate, and high-throughput field measurement of multiple ear traits. Using MAIZTRO, we evaluated the phenotypic plasticity of ear phenotypes in 3819 transgenic maize inbred lines targeting 717 genes and identified adaptive genes in plants grown in a variety of environments. Our aim was to use MAIZTRO to investigate the plasticity of ear traits using a transgenic maize inbred population and identify potential regulatory genes that perform well under different field conditions.

## Results

### High-throughput platform for phenotyping maize ear traits

**Fig. 1:**
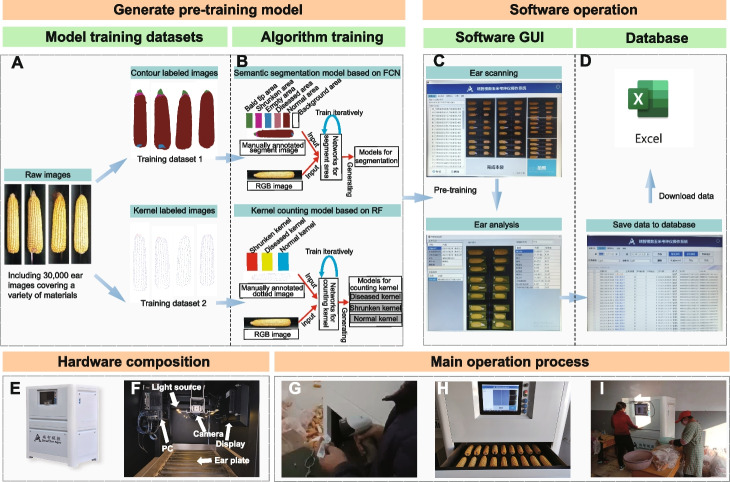
Overview of MAIZTRO software and hardware. **A** Model training datasets. **B** Algorithm training. **C** Software graphical user interface (GUI). **D** Database management. **E** Hardware exterior. **F** Hardware interior. **G** Information concerning the field plots is input by scanning a barcode. **H** Up to 18 ears can be analyzed at a time. **I** Operation requires two people: one to operate the machine and one to sort the ears. Full convolution network (FCN) and random forest (RF) models were trained using different datasets. Scanned ear images were analyzed based on the trained model, and results were stored in the database

To address the challenges of measuring maize ear traits in the field, we developed a phenotyping platform called MAIZTRO that combines all necessary software and hardware (Fig. 1). The software integrates image-processing algorithms and two trained models, including a semantic segmentation model based on the full convolution network (FCN) algorithm for dividing different kernel regions and a kernel counting model based on the random forest (RF) algorithm for counting different kernel types. Two manually labeled maize ear image datasets were used to train the FCN and RF models. First, a maize ear image dataset was generated comprising 30,000 scanned images (representing 30,000 ears) that covered inbred lines or hybrids of tropical, subtropical, and temperate maize varieties. Next, two copies of the datasets were created. In one dataset, ears were divided using boundary boxes to define areas with different kernel types, such as bare areas without kernels, diseased kernel areas, and normal kernel areas. This dataset was used to generate paired images for FCN training. In the other dataset, different colored points were used to annotate kernel types. This dataset was used to generate paired images for RF training. Cross-validation ensures the model’s generalization capabilities by allocating training and validation data at a ratio of 8:2. Finally, we used the parameters of the trained models as the basis for analysis via MAIZTRO software (Fig. 1C), which was used to operate the camera (Fig. 1F) to obtain images of up to 18 ears at a time. Traditional contour detection was used to segment a single maize ear and obtain linear measurements such as length, width, and perimeter. The trained models were then incorporated into the analysis to determine kernel type area and kernel number (Fig. 1C). The results were saved to the database, from which data could be downloaded as needed (Fig. 1D). In order to obtain complete information, photos were taken of both the front and back of each ear.

MAIZTRO uses integrated software and hardware and is highly automated with modular integration (Additional file 1: Figs. S13). The device is mobile and equipped with a display, camera, light source, PC, ear measuring disk (Fig. 1F), and handheld scanner (Fig. 1G). The scanner is used to read a barcode corresponding to the sample plot information for each ear. MAIZTRO can scan 18 ears at once and determine various kernel regions. It is designed for simple operation and easy training. With two users operating the instrument, 18 ears can be scanned approximately 700 times a day, allowing complete phenotypic characterization of approximately 12,600 ears per day (Fig. 1H, I). To evaluate the accuracy of measurements obtained by the platform, we used a number of static characteristics described by Kalantar-Zadeh [29] (see the “Methods” section for details). Evaluation of these static characteristics was based on a new data set and considered the impact of different operators, equipment, and durations on the performance prediction (Table 1). The average accuracy for number of rows per ear, number of kernels per row, ear length, ear width, and normal kernel number was greater than 95% for most static parameters (range 90.7% to 99.9%). The average accuracy for bald tip length measurements was lower, but still greater than 74% (range 74.9% to 99.9%). The number of abnormal kernels was not assessed.

**Table 1:** Static characteristics used to assess the accuracy of MAIZTRO

Static characteristic	Number of rows per ear	Number of kernels per row	Ear length	Ear width	Bald tip length	Normal kernel number
Accuracy (%)	97.5	90.7	97.9	98.1	74.9	94.1
Trueness (%)	97.3	91.5	98.0	98.3	71.9	94.7
Precision (%)	96.8	94.7	99.5	98.8	91.4	96.6
Reproducibility (%)	97.6	95.8	99.2	98.7	84.3	94.8
Technical repeatability (%)	99.95	99.98	99.99	99.6	99.97	99.99
Stability (%)	99.5	96.2	99.8	99.7	95.5	99.0

All characteristics are defined in the “Methods” section and summarized by Kalantar-Zadeh [29]

### Transgenic maize inbred population

An efficient transgene-development platform with high genetic transformation efficiency was established in the past 10 years by the Center for Crop Functional Genomics and Molecular Breeding of China Agricultural University to create maize inbred lines with specifically targeted genes using transgene technology [30, 31]. Using this platform, 3819 transgenic lines targeting 717 genes were created over a period of 2 years. These lines represent specific gene overexpression or gene knockout generated by CRISPR/Cas9 technology in the same genetic background, namely the wild-type inbred line B73-329 [32–34]. The targeted genes were selected from a set of functional maize genes defined by scientists from China Agricultural University or from cDNA libraries.

**Fig. 2:**
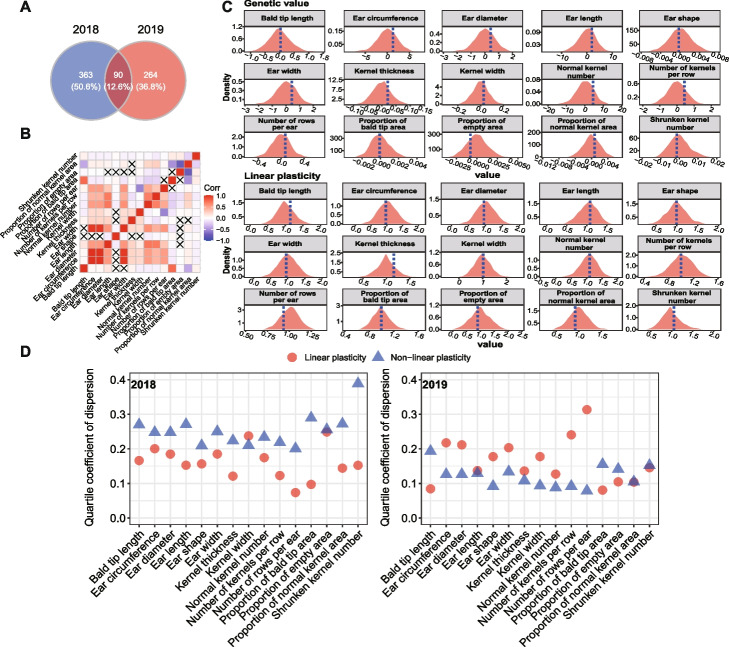
Characteristics of 717 genes involved in maize ear phenotype and phenotypic plasticity. **A** Numbers of genes studied in 2018 and 2019. **B** Correlation matrix plot of ear phenotypes of transgenic inbred lines planted in 2018 (data for 2019 are in Additional file 1: Fig. S5). **C** Distribution of genetic values and linear plasticity of phenotypes in 2018 (data for 2019 are in Additional file 1: Fig. S8). Dotted line indicates the wild-type phenotype. **D** Quartile coefficients of dispersion for linear and nonlinear plasticity of phenotypes in 2018 and 2019

In 2018 and 2019, the 3819 lines and line B73-329 were planted at several sites in the main maize production regions of China. In 2018, transgenic lines targeting 453 genes (413 overexpressed genes, 40 knockouts) were planted at Gongzhuling, Anyang, and Zhuozhou (Additional file 2: Table S2). In 2019, transgenic lines targeting 354 genes (294 overexpressed genes, 60 knockouts) were planted at Gongzhuling, Anyang, and Shangzhuang (Additional file 2: Table S3). Of the lines planted in 2018, plants harboring 90 individual transgenes were replanted and verified in 2019, for a total of 717 independent genes analyzed (Fig. 2A). Using Gene Ontology enrichment analysis to annotate and classify the functions of these genes, we identified 138 biological processes for 510 genes covering a wide range of physiological and biochemical processes (Additional file 2: Tables S4 and S5). The other 207 genes lacked Gene Ontology annotation.

### Variation and correlation among ear phenotypes

The trait values for 15 important ear phenotypes were measured for transgenic and wild-type lines using MAIZTRO. Most phenotypes conformed to a normal distribution, whereas negative-effect indices had obviously skewed distributions (Additional file 1: Figs. S1 and S2). The trait values for most ears were close to 0 for negative-effect indices, including bald tip length, proportion of bald tip area, and proportion of empty area (Additional file 1: Figs. S1 and S2).

Variations in ear phenotypes among lines were calculated across different environments using the coefficient of variation (CV), and CV distributions were plotted (Additional file 1: Figs. S3 and S4). For most phenotypes, CV values for wild-type were in the middle of the distribution (dotted lines in Additional file 1: Figs. S3 and S4), and those for transgenic lines were distributed around the values for wild-type (red shading). The CV values for kernel-related characteristics such as bald tip length, proportion of bald tip area, and proportion of empty area varied more than the other traits in both years. Ear length also varied substantially.

We next analyzed correlations among the various phenotypic characteristics (Fig. 2B; Additional file 1: Figs. S5) and found that relationships were similar in 2018 and 2019, although they were stronger in 2019. There was a strong positive correlation between normal kernel number and ear length, ear width, and number of kernels per row. The relationships between ear shape and other phenotypes were weak.

### Variation and correlation of phenotypic plasticity in ear traits

The trait values for 15 ear phenotypes for 717 genes were measured in multiple environments and analyzed using a Bayesian Finlay-Wilkinson regression (FWR) model for each phenotype [35]. This model can estimate the main effect and slope for each maize line (transgenic or wild-type), from which the residual of each observation can be calculated before estimating residual variance (i.e., nonlinear plasticity). The slope from the FWR model reflects the linear response of a genotype to the environment in the tested population (i.e., linear plasticity). A slope equal to one indicates that the transgenic line had an overall average response to the environment, whereas a slope equal to zero indicates that the line did not respond to the environment. The residual variance of each line was used as a measure of model fitting; a large residual variance indicates a lack of genetic basis for the environmental response and thus a poor linear model fit [35]. In our study, the correlations among the mean phenotype value (also called genetic value, *g*), linear plasticity (*b*), and nonlinear plasticity were analyzed (Additional file 1: Figs. S6 and S7). The *g* and nonlinear plasticity values correlated positively with bald tip length, proportion of bald tip area, and proportion of empty area, but correlated negatively with proportion of normal kernel area. For most phenotypes, the correlation between *g* and *b* values was stronger than those between *g* and nonlinear plasticity.

The dispersion of all phenotypes was evaluated using the quartile coefficient of dispersion (Fig. 2D). Most phenotypes had variable *b* values (range of quartile coefficient of dispersion, 0.1–0.3, Fig. 2D), indicating diverse plasticity mechanisms among the different genotypes. Dispersion values were similar in 2018 and 2019. Dispersion of *b* values was lower for bald tip length, ear length, and proportion of bald tip, but higher for ear diameter and kernel width (Fig. 2D). Nonlinear plasticity had a higher degree of dispersion than *b* values in 2018 but a similar degree of dispersion in 2019 (Fig. 2D). The *g* and *b* values (Fig. 2C, Additional file 1: Figs. S8) were nearly normally distributed in both years, with wild-type almost always appearing in the middle of the distribution (Fig. 2C, Additional file 1: Figs. S8).

### Screening of target genes

**Fig. 3:**
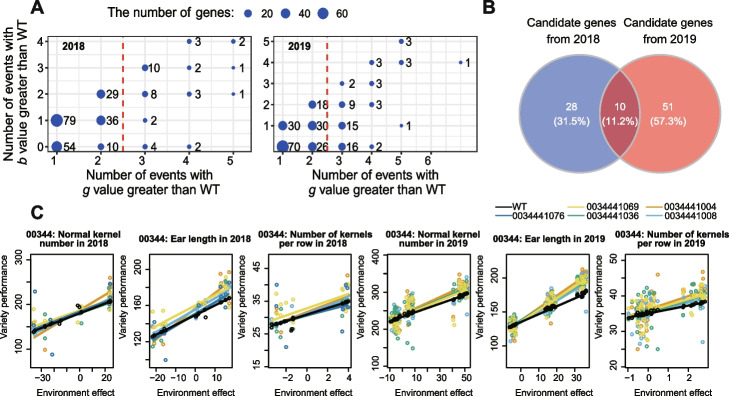
Screening of transgenic lines/genes. **A** Numbers of genes in the classification of candidate genes. WT: wild-type. **B** Number of genes for which the genetic value of at least three lines was higher than that of wild-type. **C** Plasticity of kernel number, ear length, and number of kernels per row for gene 00344 (*GRMZM2G012891*) in 2018 and 2019. Data for other candidate genes are presented in Additional file 1: Figs. S15-S17

**Fig. 4:**
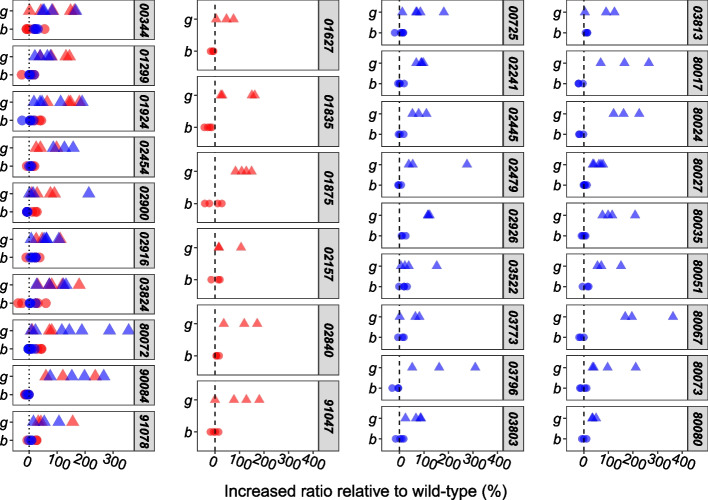
Phenotypic plasticity for 34 lines with high plasticity. *g*: mean phenotypic value, *b*: linear plasticity, vertical dotted line: plasticity of wild-type. Red dots and triangles represent planting in 2018; and blue dots and triangles represent planting in 2019

The phenotypic plasticity of plants using the FWR method is represented by the *g* and *b* values [7, 11]. The plasticity characteristics of the transgenic lines are shown as plots of *g* versus *b* (Additional file 1: Figs. S9 and S10), where the performance of different transgenic lines (red dots) can be divided into four regions based on the performance of wild-type (blue lines). To screen for genes that may regulate phenotypic plasticity, we selected kernel number per ear (here, normal kernel number) as an ear phenotype that is directly related to yield. For this trait, a higher *g* value means a higher kernel number per ear in different environments [7, 11]. Thus, we first selected lines with *g* values greater than that of wild-type. In the selected lines, we determined the number of events (lines) for each retained gene in which the *g* and *b* values were greater than those of wild-type and counted genes corresponding to different events (Fig. 3A). We then selected candidate genes with *g* values greater than that of wild-type in at least three different lines. This resulted in 38 and 61 candidate genes from 2018 and 2019, respectively, including 10 genes from both 2018 and 2019 (Fig. 3B). Finally, we used the Bayesian FWR model to evaluate the plasticity of these genes with respect to three ear phenotypes: kernel number, ear length, and number of kernels per row. In the FWR model, the slope of the line reflects the linear plasticity of events of these genes, the position of the line (which depends on *g*) reflects the phenotypic variation of events of these genes, and the black line represents wild-type; other colors represent the plasticity of different events of a single gene (Fig. 3C, Additional file 1: Figs. S15-S17). With these selected genes, we continued to filter those with small advantages in which the *g* value was only slightly greater than that of wild-type. Ultimately, we identified 34 candidate regulatory genes, including 10 genes from 2018 and 2019, 6 genes from 2018 only, and 18 genes from 2019 only (Additional file 2: Table S6). Figure 4 shows the changes in *g* and *b* values relative to wild-type for all 34 genes. The material class and corresponding gene information for the 34 identified genes are summarized in Table 2 and Additional file 2: Table S6.

**Table 2:** Information on candidate regulatory genes associated with excellent plasticity in 2018 and 2019

Year	Material ID	Material class	Gene ID	Chromosomal localization
2018–2019	00,344	OE	*GRMZM2G012891*	Chr7
2018–2019	01,299	OE	*GRMZM2G093195*	Chr9
2018–2019	01,924	OE	*GRMZM2G328785*	Chr6
2018–2019	02,454	OE	*GRMZM2G333183*	Chr10
2018–2019	02,900	OE	*GRMZM2G164475*	Chr3
2018–2019	02,916	OE	*GRMZM2G142409*	Chr6
2018–2019	03,824	OE	*GRMZM2G178795*	Chr4
2018–2019	80,072	Cas9	*GRMZM2G134708_T01*	Chr4
2018–2019	90,084	Cas9	*GRMZM2G077278_T01*	Chr6
2018–2019	91,078	OE	*AT4G17870.1*	Chr4 in Arabidopsis
2018	01,627	OE	*GRMZM2G123387*	Chr2
2018	01,835	OE	*GRMZM2G150796*	Chr7
2018	01,875	OE	*GRMZM2G360589*	Chr5
2018	02,157	OE	*GRMZM2G111906*	Chr6
2018	02,840	OE	*GRMZM2G470942*	Chr8
2018	91,047	OE	*GRMZM2G446858*	Chr3
2019	00,725	OE	*GRMZM2G009913*	Chr5
2019	02,241	OE	*GRMZM2G034622*	Chr6
2019	02,445	OE	*GRMZM2G312521*	Chr4
2019	02,479	OE	*GRMZM2G414460*	Chr7
2019	02,926	OE	*GRMZM2G347361*	Chr2
2019	03,522	OE	*GRMZM2G145879*	Chr6
2019	03,773	OE	*GRMZM2G479318*	Chr1
2019	03,796	OE	*MGG_05056*	Chr3 in *Pyricularia oryzae*
2019	03,803	OE	*MGG_06559*	Chr4 in *Pyricularia oryzae*
2019	03,813	OE	*MGG_10492*	Chr2 in *Pyricularia oryzae*
2019	80,017	Cas9	*GRMZM2G368838_T01*	Chr2
2019	80,024	Cas9	*GRMZM2G090435_T01*	Chr5
2019	80,027	Cas9	*GRMZM2G014136_T01*	Chr3
2019	80,035	Cas9	*GRMZM2G130442_T01*	Chr4
2019	80,051	Cas9	*GRMZM2G126505_T01*	Chr4
2019	80,067	Cas9	*GRMZM2G099425_T01*	Chr2
2019	80,073	Cas9	*GRMZM2G090241_T01*	Chr4
2019	80,080	Cas9	*GRMZM2G004483_T01*	Chr9

*OE *overexpression of a specific gene, *Cas9 *knockout of a specific gene. Gene annotation information is reported in Additional file 2: Table S6

For plants harboring most of these genes, plasticity was similar for normal kernel number, ear length, and number of kernels per row. The slopes for the transgenic lines were equal to or greater than those of wild-type, indicating that these lines will perform better than wild-type in a new environment. For these genes, *g* values varied more than *b* values. Furthermore, the increase in *b* values relative to wild-type was concentrated around 0, whereas the increase in *g* values relative to wild-type was significantly greater than 0 (Fig. 4). In more suitable environments, the phenotypic differences between the 34 identified lines and wild-type were more pronounced (Additional file 1: Figs. S15-S17).

## Discussion

Transgene technology has been widely used in crop breeding and gene discovery and has produced many transgenic lines with clear advantages for laboratory research [15]. However, only a few publications have reported that genetic engineering can produce transgenic crops with higher yields than conventional breeding under field conditions [16], although changes in natural gene expression in maize can produce substantial positive changes in complex traits [16]. By inducing gene overexpression or knockout, specific transgenic lines can be created and cultivated in multiple field environments for the purpose of phenotype comparison with wild-type plants and identification of genes responsible for phenotypic differences. Obtaining transgenic lines covering a variety of biological processes in a short time is key to maize improvement based on gene technology [36]. To this end, an efficient and standardized transgenic platform was established [30, 37] and utilized in the present study. We used 3819 transgenic lines targeting 717 genes and revealed 138 Gene Ontology biological processes involved in a variety of stress-responsive genes. This transgene dataset enabled us to evaluate the field performance of multiple genes at the same time and screen potential regulatory genes.

A faster and better phenotypic measurement system is necessary to facilitate breeding [38]. Currently, there is no good tool for high-throughput field measurement of the number of kernels per ear or related phenotypes [22–25]. To accurately and efficiently achieve comprehensive phenotyping of maize ears at various experimental sites, MAIZTRO was developed and has been installed at our experimental sites. MAIZTRO shows obvious advantages compared with other measurement systems. MAIZTRO realizes the integration of software and hardware, forming a complete ear measurement workflow and mature products that are different from some simple image acquisition platforms [28, 39]. The platform is simple to operate without complex knowledge after simple training and does not require complex configuration as with the system of Miller et al. [27]. Compared to Warman et al. [28], which can measurement only one ear at a time and focuses on kernel phenotype, MAIZTRO offers high-efficiency and comprehensive measurement of 15 ear traits from 18 ears at a time. Therefore, using this platform, we can measure all ears in a plot to eliminate sampling errors. MAIZTRO is especially suitable for use in the field because the closed setting prevents interference from environmental factors, which are more difficult to eliminate using the simple devices of Makanza et al. [39] and Warman et al. [28]. Therefore, MAIZTRO is suitable for multi environment assessment of ear phenotype of a large number of materials in the field.

Some plant phenotypes are more plastic than others [7, 11], and such variations in phenotypic plasticity reflect gene-environment interactions. Maize yield depends on development of the ear and is strongly impacted by phenotypic variation of ear traits [39]. In our study, most phenotypic values and their variation showed a normal distribution. Wild-type values were always in the middle of the distribution, indicating that the targeted genes affected phenotypic variation in different ways and to varying degrees. In our analysis using the FWR model, the *b* value variation (linear plasticity, quartile coefficient of dispersion) was mostly in the range of 0.1 to 0.3 and was similar in 2018 and 2019; nonlinear plasticity showed a similar trend, although it differed between the 2 years (Fig. 2D). These results suggest that the impact of phenotypic plasticity on maize ears was essentially uniform across the transgenes analyzed. The distributions of *g* and *b* values were similar to those of phenotypic value and variation (a normal distribution with wild-type in the middle), indicating that the genes studied also have different effects on phenotypic plasticity. Our data support the idea that phenotypic plasticity is genetically regulated [7, 10, 11] and that phenotypic plasticity of maize ears can be altered by transgene technology targeting specific genes. In addition, similar to the findings of Kusmec et al. [7], we found a correlation between mean phenotype values and linear plasticity in most ear phenotypes, although the correlation for ear phenotypes was relatively weak compared with the maize growth phenotypes described by Kusmec et al. [7].

Our screen identified 34 candidate genes in the 2018 and 2019 planting years that had led to greater normal kernel number compared with wild-type in different environments and also had similar or greater plasticity (Additional file 1: Figs. S15-S17). Ear length and number of kernels per row, which are significantly related to normal kernel number, showed the same trend. This suggests that the 34 genes increase normal kernel number by regulating ear length and number of kernels per row and confer advantages over wild-type for response to environmental effects. The Additive Main Effects and Multiplicative Interaction (AMMI) model and coefficient of variation (CV) combined with the mean normal kernel number were used to validate the 34 identified genes and revealed that the mean and stability of normal kernel number for these 34 genes was significantly higher than for wild-type (Additional file 1: Figs. S11&S12).

Among the 34 candidate regulatory genes identified, *GRMZM2G414460* and *GRMZM2G347361* have previously been associated with days to silk and cob diameter, respectively, in a genome-wide association study [7]. In addition, *GRMZM2G012891*, *GRMZM2G368838_T01*, and *GRMZM2G090435_T01* are related to temperature adaptation (Additional file 2: Table S6). Our experimental planting regions span from 36° to 43° north latitude and thus represent different climatic conditions (Additional file 2: Table S1). *GRMZM2G333183* is strongly associated with salt stress [40, 41], *GRMZM2G004483* and *GRMZM2G178795* with maize photoperiodic flowering and improved maize adaptation to high latitudes [42–44], and *GRMZM2G134708* with maize adaptation to cold stress [45, 46]. Although these reports did not directly mention ear phenotypes, they all contribute to the environmental adaptation of maize, potentially affecting its response to the environment. In addition, 4 of the 34 identified genes (*GRMZM2G178795*, *GRMZM2G123387*, *GRMZM2G111906*, and *GRMZM2G126505*) appeared in the selected genes during maize domestication and improvement [47]. Interestingly, *GRMZM2G077278* was modified by both gene knockout and overexpression simultaneously in 2018, and knockout of *GRMZM2G077278* increased the *g* values of normal kernel number, whereas overexpression significantly reduced *g* values (Additional file 1: Figs. S14).

Three hypotheses have been proposed to explain the genetic underpinnings of phenotypic plasticity, including overdominance, pleiotropy, and the structural (or regulatory) gene model [7, 11]. Some studies have shown that pleiotropic and epistatic quantitative trait loci may play a key role in the phenotypic plasticity of maize inbred lines [7, 48]. The pleiotropy model argues that specific genes that affect mean phenotypic values and are differentially sensitive to the environment are responsible for phenotypic plasticity [49]. The structural (or regulatory) gene model suggests that plasticity is a consequence of the regulation of genes underlying the mean phenotypic value by other genes that transduce environmental stimuli [7]. In our study, there was a moderate correlation between *g* and *b* values (Additional file 1: Figs. S6 and S7), and, in most cases, higher *g* values correlated with higher *b* values. This indicates an internal relationship between plasticity and genetic value, as previously reported by Kusmec et al. [7], and strongly supports the structural/regulatory gene model.

Phenotypic plasticity is defined as the phenotypic variation of a trait when an organism is exposed to different environments, which can well reflect the stability of specific phenotypes of materials. We can use this method to evaluate the yield stability of different materials under different environments. In breeding, we hope to obtain a material that can not only increase yield in a high-yield environment compared with the reference material, but can also increase yield in a low-yield environment, thus improving the overall stability of the material. Our method can be used to identify genes that contribute to yield increase under high- and or low-yield conditions. This has important applications for enriching germplasm materials, improving breeding, and recommending specific materials or genes for use in different geographical regions.

## Conclusions

MAIZTRO was used to screen 3819 transgenic maize inbred lines covering 717 genes, allowing the identification of 34 candidate genes that contribute to yield stability and are involved in multiple biological processes. Future investigation is needed to determine the regulatory mechanisms by which these genes contribute to phenotypic plasticity. Only a high-throughput phenotypic platform suitable for large-scale field measurement can meet the needs of evaluating the performance of a large number of materials in multiple areas for many years to determine new high-quality germplasm materials. The MAIZTRO platform, the transgenic inbred population, and the 34 identified genes provide new avenues for developing maize varieties with high and stable yields in a variety of regions and in the face of changing climates.

## Methods

### Sample material and planting

The maize lines used in this study included 3819 transgenic inbred lines targeting 717 genes and one wild-type inbred line (B73-329) as a reference (Additional file 2: Table S2 and S3). Each transgenic line facilitated single-gene overexpression or knockout relative to the wild-type line. The recipient for all transgenic lines was B73-329, and targeted genes were overexpressed or knocked out using CRISPR/Cas9 technology [32, 34].

Transgenic kernels were planted in different environments in two different years, including 2217 transgenic lines (453 genes) in 2018 and 1602 transgenic lines (354 genes) in 2019. Lines planted in 2018 included 40 gene knockouts and 413 overexpressed genes (Additional file 2: Table S2). Lines planted in 2019 included 57 gene knockouts and 297 overexpressed genes (Additional file 2: Table S3). The planting sites in 2018 were Gongzhuling (N 43.5°, E 124.8°) in Jilin Province; Anyang (N 36.0°, E 114.1°) in Henan Province; and Zhuozhou (N 39.5°, E 115.8°) near Beijing. The planting sites in 2019 were Gongzhuling, Anyang, and Shangzhuang (N 40.1°, E 116.2°) in Beijing. In each planting year, the same lines were planted at each of the three sites. Kernels were planted according to the same block design at each site, and microenvironments were divided by block (Additional file 2: Table S2 and S3). Additional file 2: Table S1 summarizes the meteorological characteristics of the sites.

### Analysis of ear phenotypes

In each year, all three sites were harvested simultaneously when 95% of ears reached full maturity. MAIZTRO was then used to analyze the following 15 ear phenotypes for all ears (Fig. 1): bald tip length, ear circumference, ear diameter, ear length, ear shape, ear width, kernel thickness, kernel width, normal kernel number, shrunken kernel number, number of kernels per row, number of rows per ear, proportion of bald tip area, proportion of empty area, and proportion of normal seed area. Ear shape was calculated as the ratio of ear width at one-third the length of the ear from the tip (first third) to ear width at one third the length of the ear from the base (last third). For the last three traits, proportion was calculated as the ratio of total area ranging from 0 to 1. For all traits, we filtered out outliers exceeding 1.5 times the interquartile range in the population prior to analysis.

### Model training and verification of MAIZTRO

MAIZTRO calculates parameters primarily related to ear contour and kernel type using the FCN and RF algorithms, which require a large amount of data for training to obtain ideal performance (Fig. 1). We collected images of 30,000 ears from inbred lines and hybrids of tropical, subtropical, and temperate maize varieties covering a rich ear diversity. Based on these images, we manually created two labeled image sets (Fig. 1). The first set consisted of pairs of RGB (red, green, blue) images and manually labeled segmentation images and was used to train the FCN model. The second set consisted of a series of paired RGB images and manually labeled images in which points of different colors represented different kernel types (for example, blue for normal kernels) and was used to train the kernel-counting RF model.

Six indicators were used to evaluate the measurement performance of the platform for major phenotypes including number of rows per ear, number of kernels per row, ear length, ear width, bald tip length, and normal kernel number [29].

*Accuracy* was defined as the agreement between a single measured value and the real value. A sample of 200 ears was measured using MAIZTRO to give the measured value (MV) and manually to give the real value (RV). The accuracy for each ear was calculated as:

$${\text{Accuracy}} = 100 - \left( {\frac{{{\text{MV}} - {\text{RV}}}}{{{\text{RV}}}} \times 100} \right)$$

The accuracy for the platform was then calculated as the average accuracy from 200 ears.

*Trueness* was defined as the agreement between an average measured value from multiple measurements and the real value. The same 200 ears were measured one time manually and three times using MAIZTRO. The same person measured each ear all three times using MAIZTRO to ensure consistency of the process (i.e., measuring the ear, removing it, and measuring it again). Trueness was calculated as the accuracy of the average of the three measurements.

*Precision* was defined as the repeatability of multiple measurements under the same user-platform conditions without regard to real value. The same 200 ears were measured three times by the same person using MAIZTRO with the same process. The CV was calculated for each ear and then the average CV of all 200 ears was calculated.

*Reproducibility* was defined as the repeatability of multiple measurements under different user-platform conditions. For this, 100 ears were measured by five different people operating five different platforms. Each ear was measured five times total. The CV of each ear was calculated and then the average CV of all 100 ears was calculated.

*Technical repeatability* was defined as the ability of a single instrument to produce the same results across several measurements. Using the same platform, 18 ears were measured 1000 times by the same person with fixed placement (i.e., without being removed). The CV was calculated for each ear and then the average CV was calculated.

*Robustness* was defined as the ability of a sensing system to produce the same output value when measuring the same object over a period of time. The same 18 ears were measured once a day for 10 days. The CV was calculated for measurements made on different days for each ear, and then the average CV was calculated.

### Stability analysis

Phenotype plasticity of each transgenic inbred line and the wild-type line was estimated across environments by applying the FWR model with the FW package in R software [35]. For this model, the individual phenotype observed in an environment could be expressed as:

$${y_{ij}} = {\upmu } + {g_i} + \left( {1 + {b_i}} \right){h_j} + {{\upvarepsilon }_{{\text{ij}}}}$$

where *y*_*ij*_ is the phenotype of line *i* (either transgenic or wild-type) collected in the *j*th environment, *μ* is the population mean, *g*_*i*_ is the main effect of line *i* and reflects the estimated value of the mean phenotype, (1 + *b*_*i*_) is the linear plasticity of line *i* over the environments, (1 + *b*_*i*_) *h*_*j*_ indicates the phenotypic change of line *i* in a given environment, and ɛ_*ij*_ is an error term; variance was recorded as a measure of the nonlinearity of the response to the environment [7, 11].

## Supplementary Information

**Additional file 1: Figure S1.** Phenotypic variation of ears from transgenic inbred lines planted in 2018. **Figure S2.** Phenotypic variation of ears from transgenic inbred lines planted in 2019. **Figure S3.** Distribution of the coefficient of variation for transgenic inbred lines planted in 2018. **Figure S4.** Distribution of the coefficient of variation for transgenic inbred lines planted in 2019. **Figure S5.** Correlation matrix plot of phenotypic ear characteristics of transgenic inbred lines planted in 2019. **Figure S6.** Genetic correlations among mean phenotype values, linear plasticity, and nonlinear plasticity for transgenic inbred lines planted in 2018. **Figure S7.** Genetic correlations among mean phenotype values, linear plasticity, and nonlinear plasticity for transgenic inbred lines planted in 2019. **Figure S8.** Distribution of genetic values and linear plasticity of phenotypes for transgenic inbred lines planted in 2019. **Figure S9.** Phenotypic plasticity of transgenic lines planted in 2018. **Figure S10.** Phenotypic plasticity of transgenic lines planted in 2019. **Figure S11.** Analyzing the performance of 16 candidate regulatory genes screened in 2018 based on AMMI model, coefficient of variation (CV), and average phenotypic value. **Figure S12.** Analyzing the performance of 28 candidate regulatory screened in 2019 based on AMMI model, coefficient of variation (CV), and average phenotypic value. **Figure S13.** Flow chart of relationship between different layers of MAIZTRO. **Figure S14.** Effect of overexpression and knockout of *GRMZM2G077278* on phenotypic plasticity for normal kernel number in 2018. **Figure S15.** Plot from FWR analysis of genes that performed well and were present in lines planted in both 2018 and 2019 (*n* = 10 genes). **Figure S16.** Plot from FWR analysis of genes that performed well and were present in lines planted only in 2018 (*n* = 6 genes). **Figure S17.** Plot from FWR analysis of genes that performed well and were present in lines planted only in 2019 (*n* = 18 genes).

**Additional file 2: Table S1.** Meteorological characteristics of experimental sites. **Table S2.** Transgenic lines planted in 2018. **Table S3.** Transgenic lines planted in 2019. **Table S4.** Gene Ontology enrichment analysis of all 717 genes from 2018 and 2019. **Table S5.** Classification of Gene Ontology biological processes. **Table S6.** Information on candidate regulatory genes associated with good plasticity.

**Additional file 3.** Review history.

## Data Availability

All analysis results supporting the findings in this study are available within this article and its additional files. The ears phenotypic data of the transgenic maize inbred population and the related scripts used in this study are deposited under a GPL-3.0 license in Github [50]. The ear image data are stored in https://doi.org/10.5281/zenodo.7796696 [51]. The scripts and data used in this study are available under a GPL-3.0 license in Github: https://github.com/liumiguo/paper_ear_pp_code.git [50] and in Zenodo https://doi.org/10.5281/zenodo.7792895 [52]. The data generated during the analysis are also stored in Github [50].
