# Supplementary material for: Identifying yield-related genes in maize based on ear trait plasticity
Source: Genome Biol. 2023 Apr 25;24:94. doi: 10.1186/s13059-023-02937-6 (PMC10127483; doi:10.1186/s13059-023-02937-6)

**Additional file 1:**


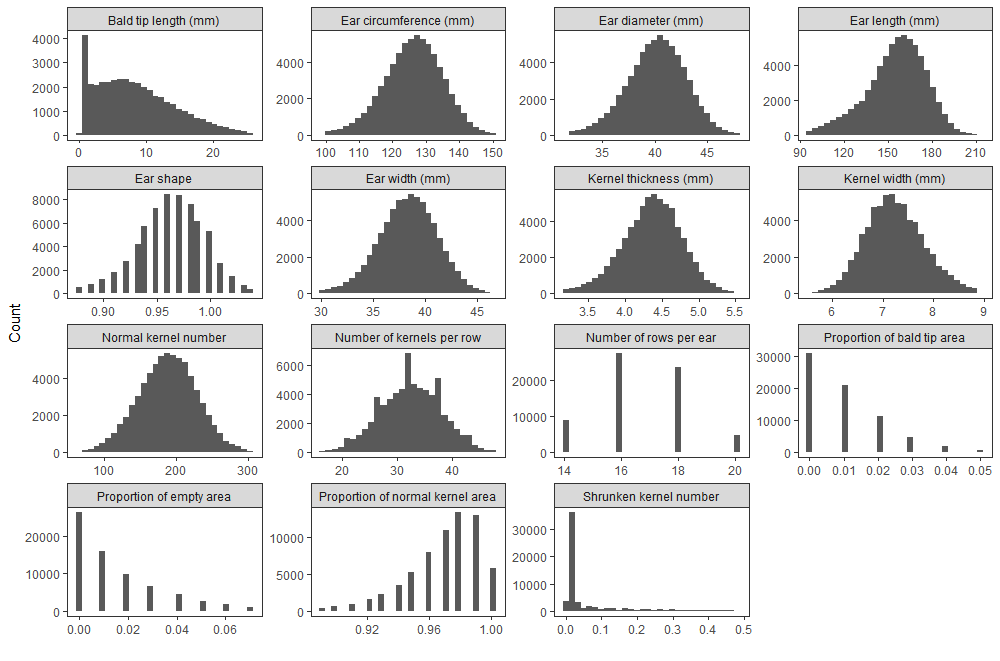


**Fig. S1** Phenotypic variation of ears from transgenic inbred lines planted in 2018. See Methods for calculation of ear shape and proportion of areas.


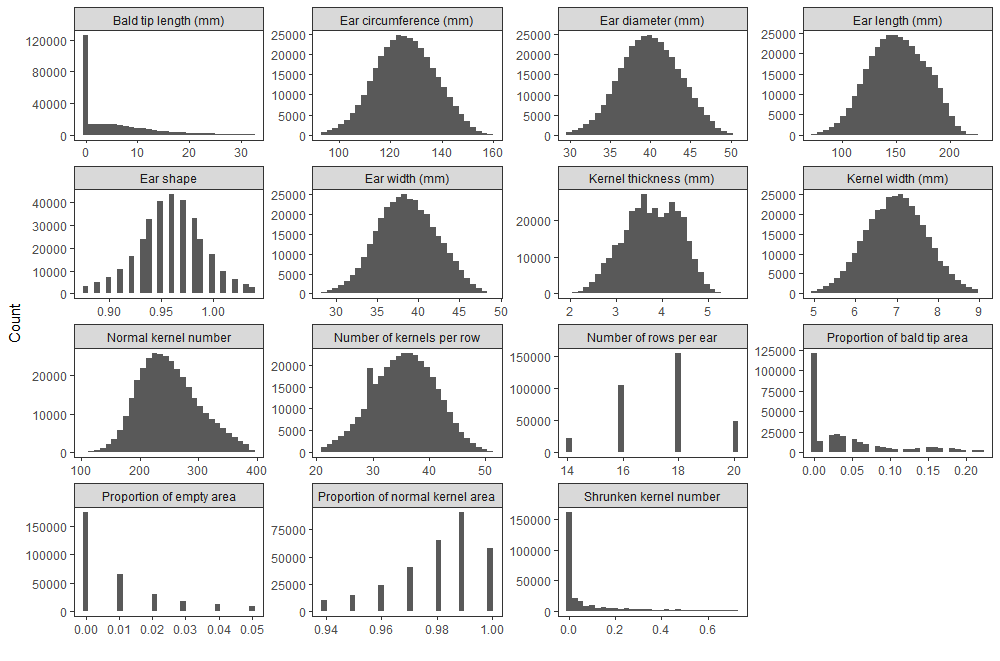


**Fig. S2** Phenotypic variation of ears from transgenic inbred lines planted in 2019.


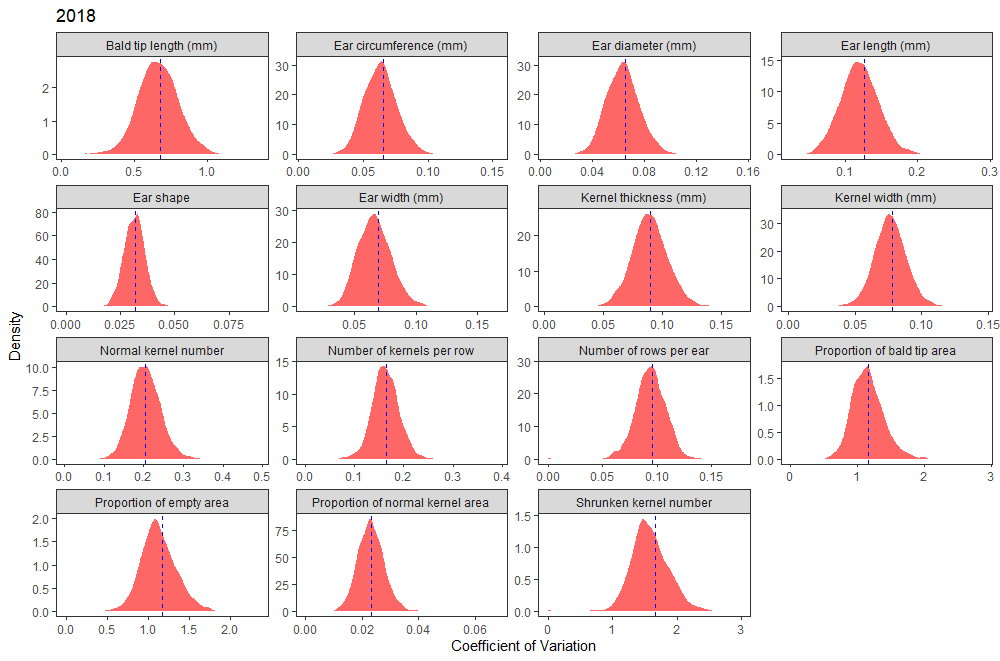


**Fig. S3** Distribution of the coefficient of variation for transgenic inbred lines planted in 2018. Vertical dotted line indicates coefficient of variation of wild-type.

**
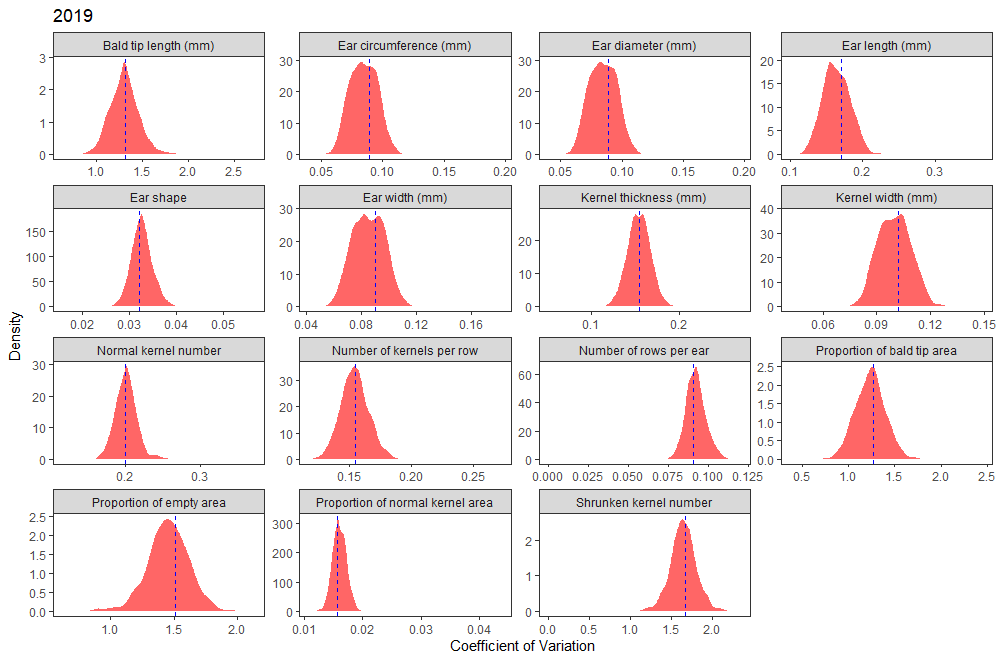
**

**Fig. S4** Distribution of the coefficient of variation for transgenic inbred lines planted in 2019. Vertical dotted line indicates coefficient of variation of wild-type.


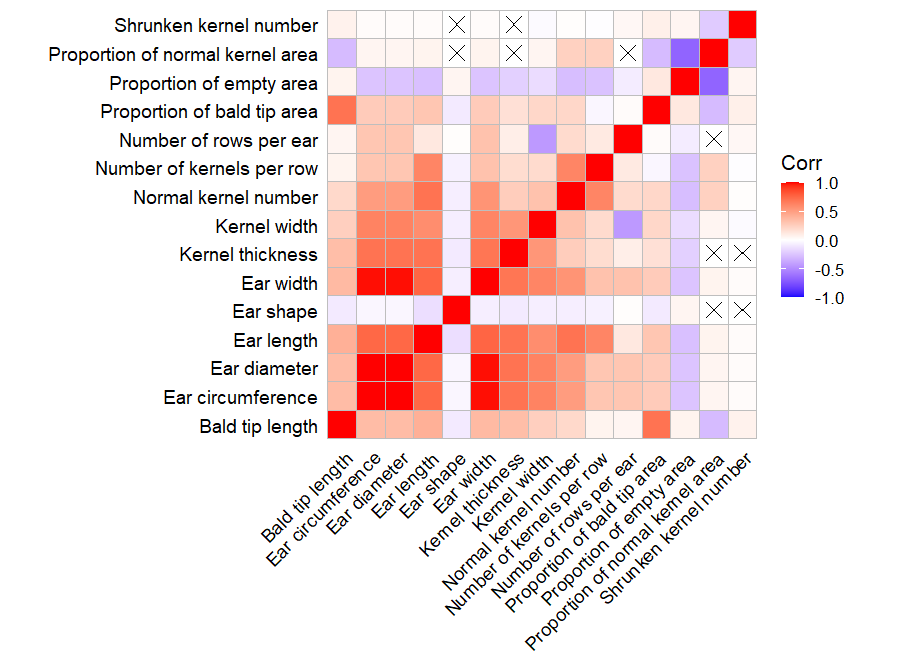


**Fig. S5** Correlation matrix plot of phenotypic ear characteristics of transgenic inbred lines planted in 2019.

**
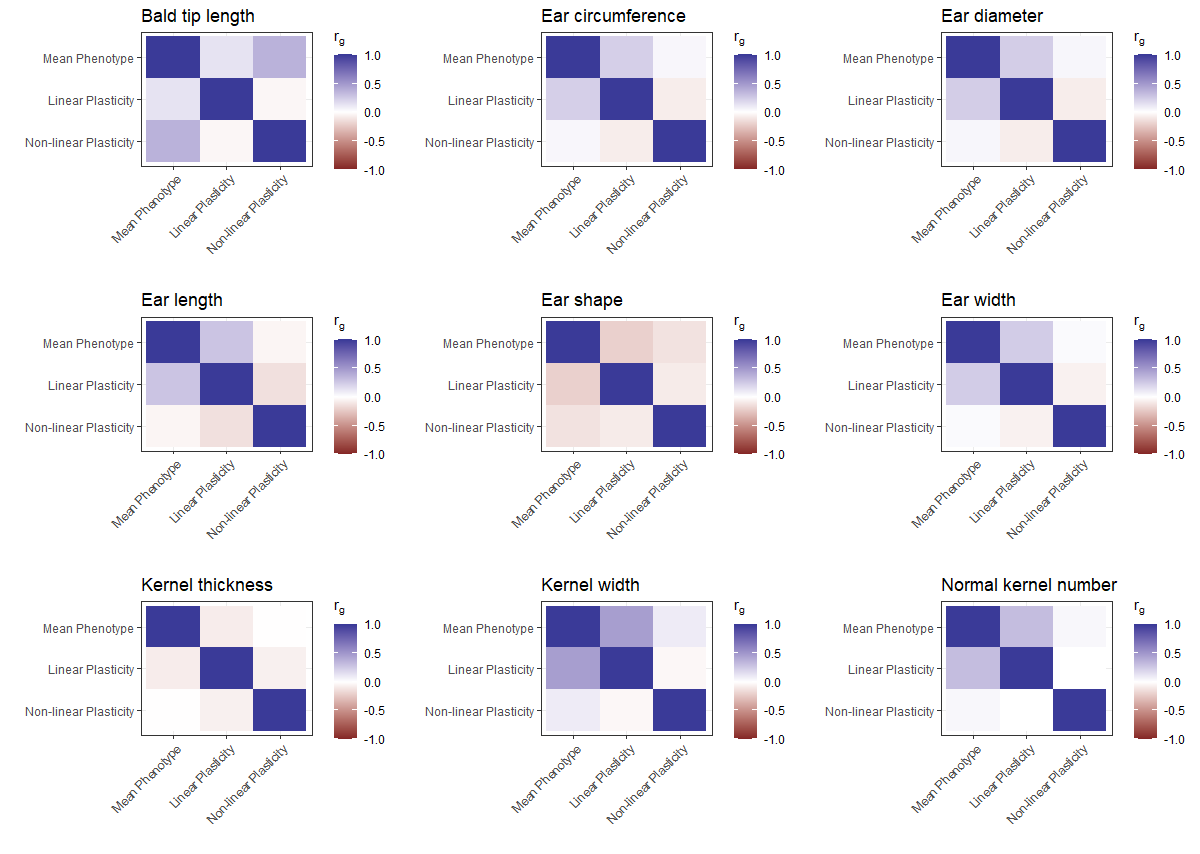
**

**
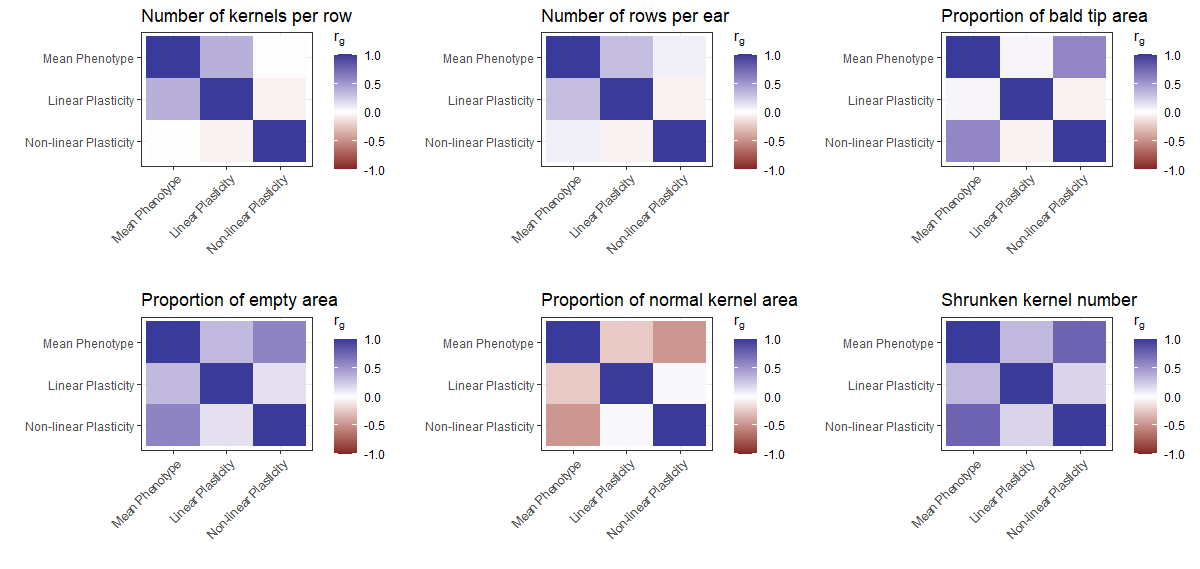
**

**Fig. S6** Genetic correlations among mean phenotype values, linear plasticity, and nonlinear plasticity for transgenic inbred lines planted in 2018.


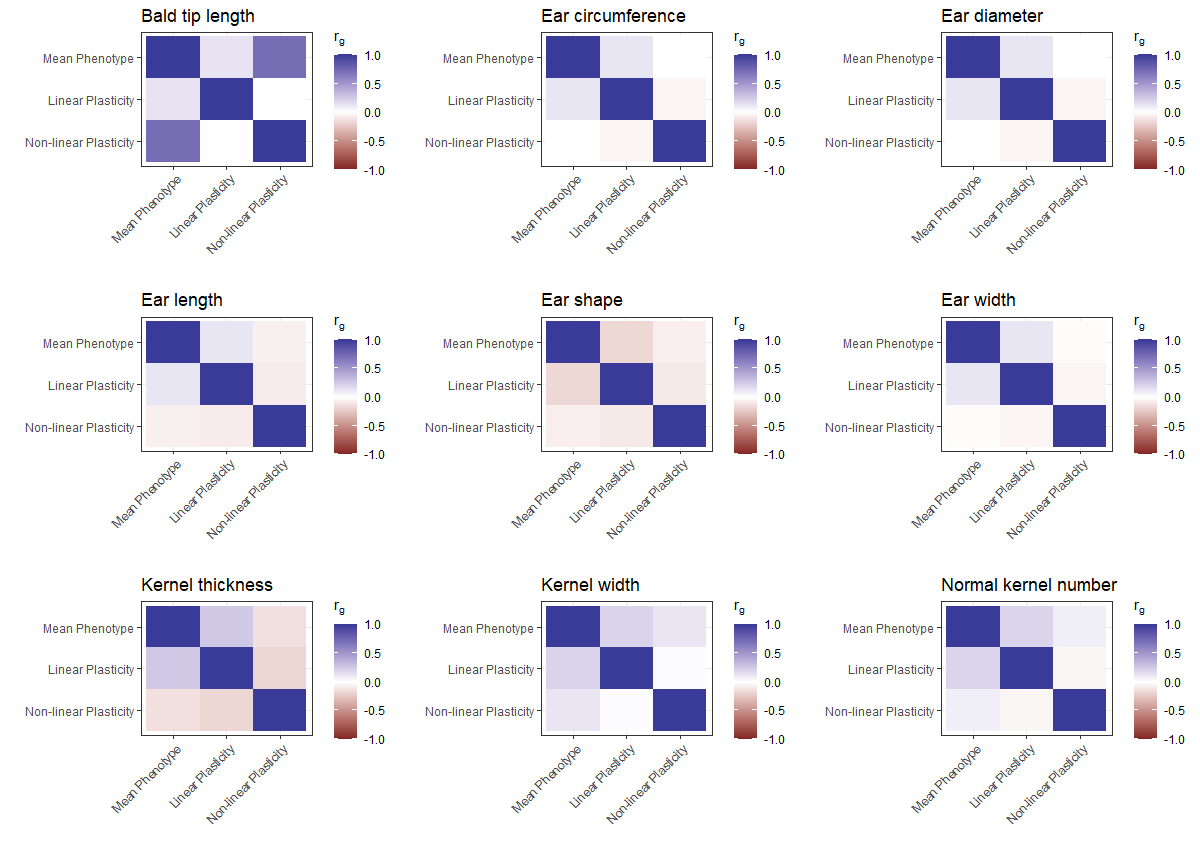


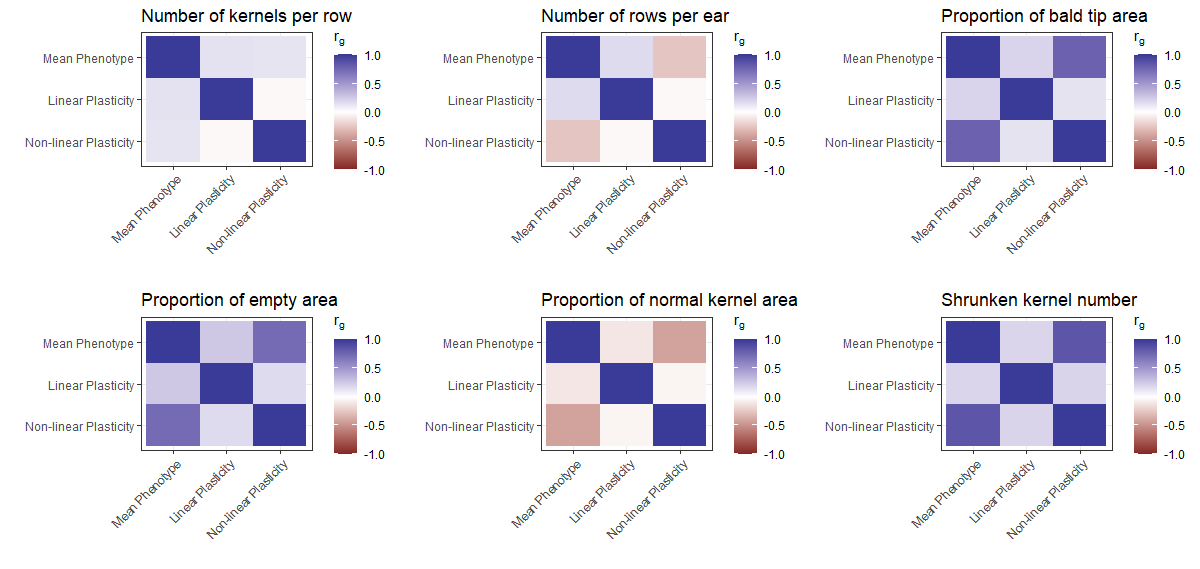


**Fig. S7** Genetic correlations among mean phenotype values, linear plasticity, and nonlinear plasticity for transgenic inbred lines planted in 2019.

**
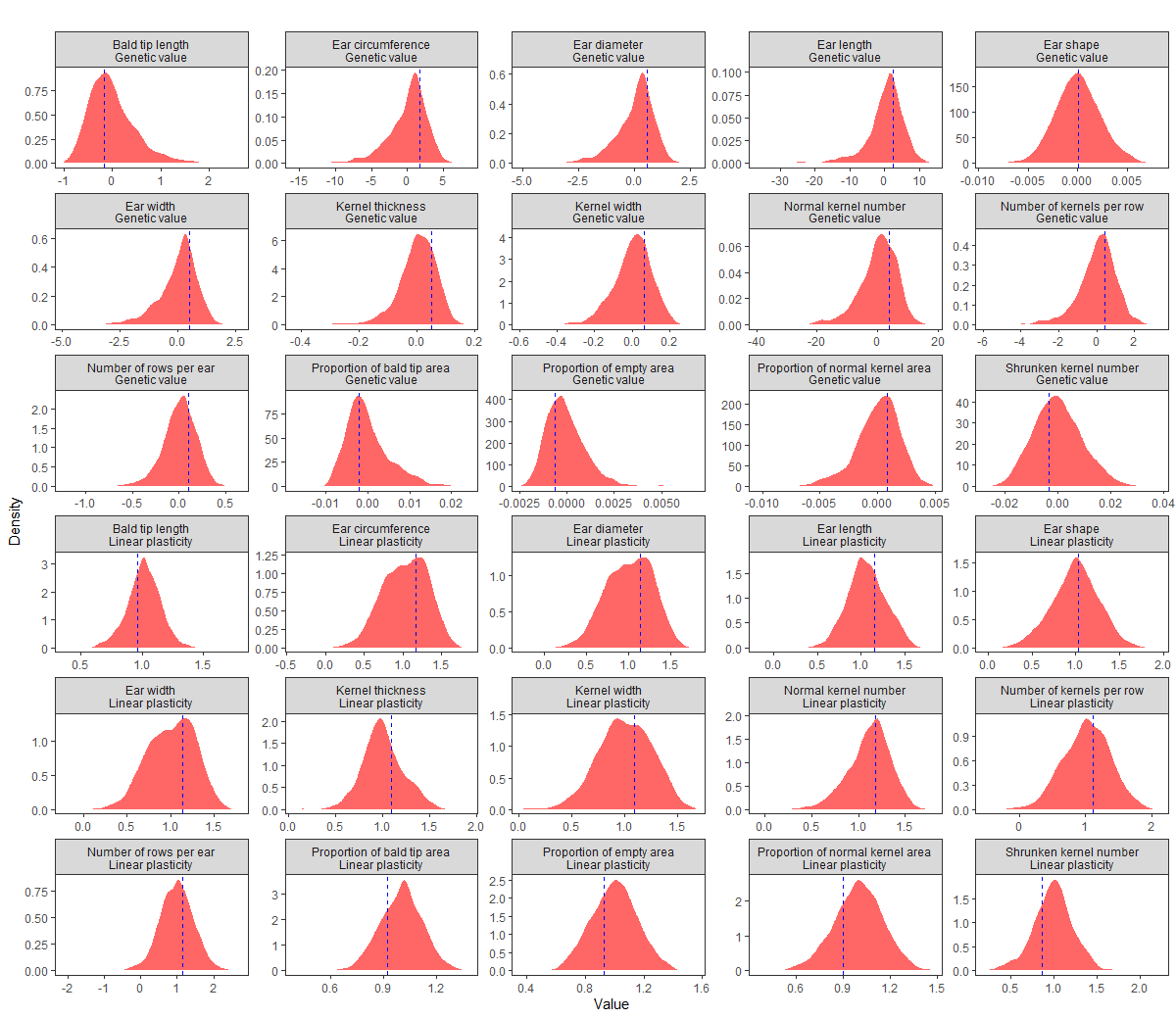
**

**Fig. S8** Distribution of genetic values and linear plasticity of phenotypes for transgenic inbred lines planted in 2019. The vertical dotted line indicates the phenotypic plasticity of wild-type.


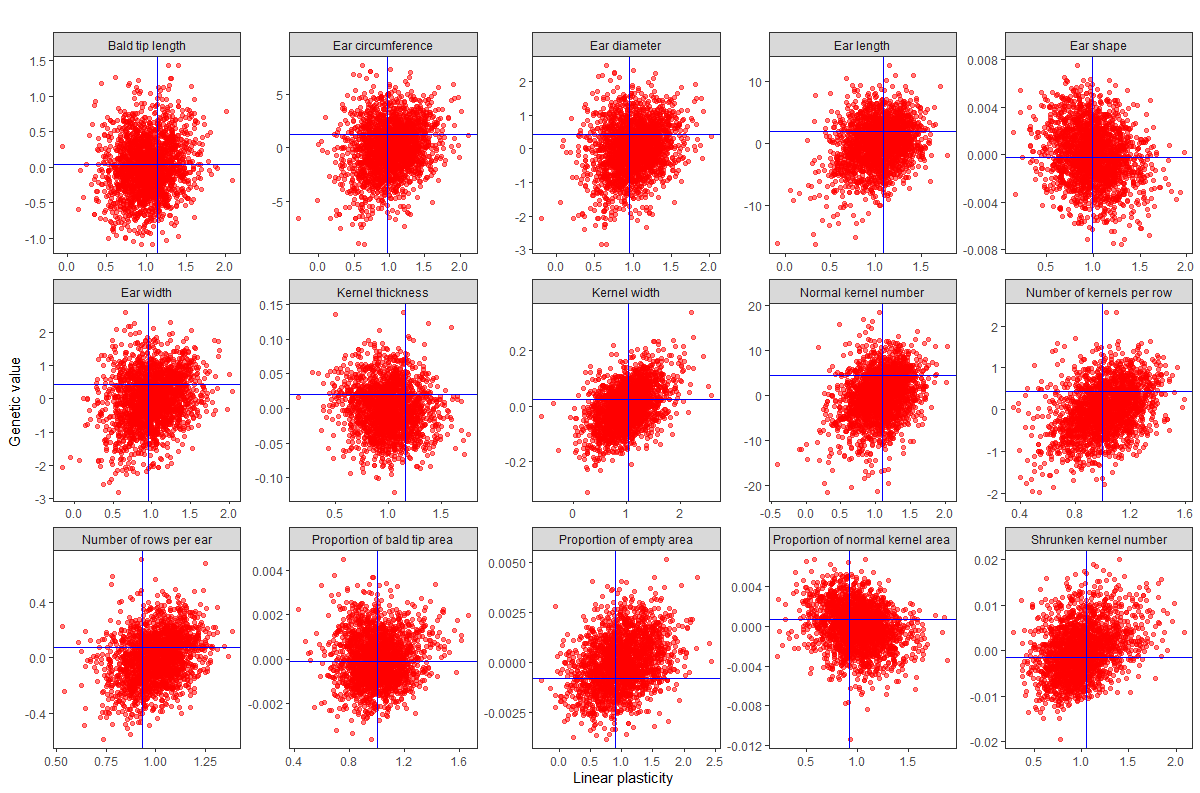


**Fig. S9** Phenotypic plasticity of transgenic lines planted in 2018. Horizontal and vertical blue lines indicate phenotypic plasticity of wild-type.


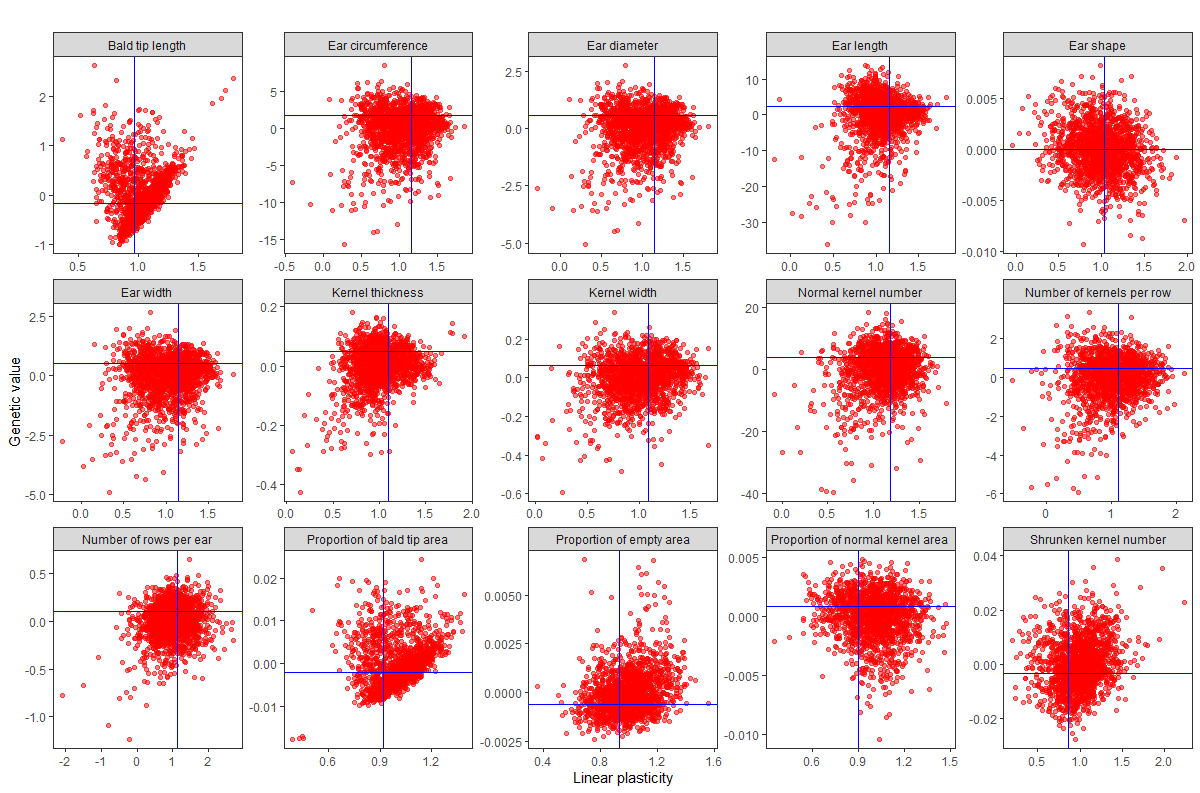


**Fig. S10** Phenotypic plasticity of transgenic lines planted in 2019. Horizontal and vertical blue lines indicate phenotypic plasticity of wild-type.


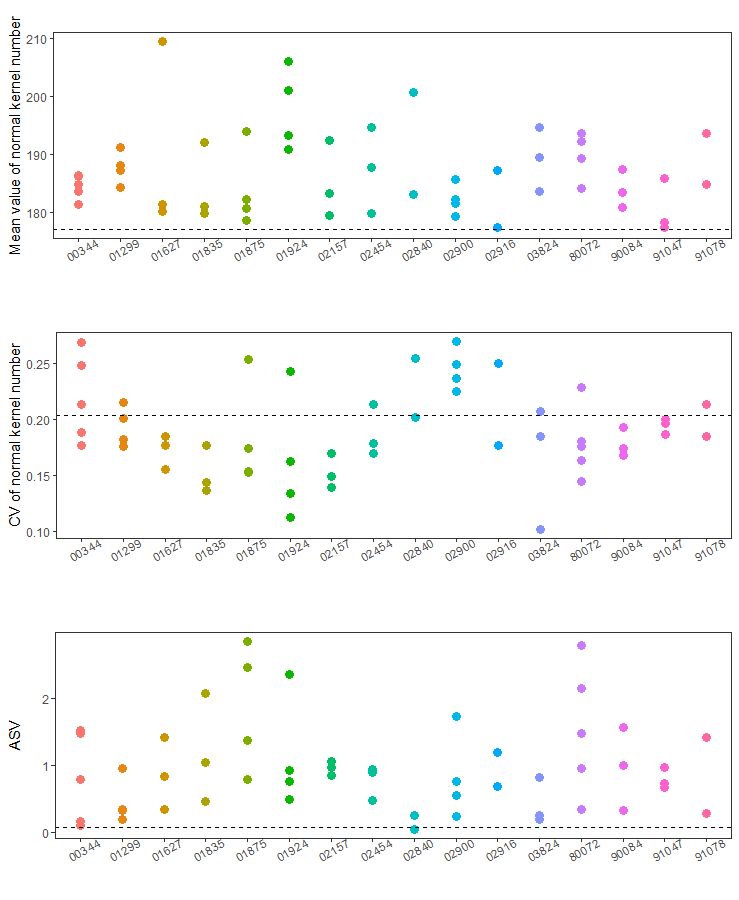


**Fig. S11** Analyzing the performance of 16 candidate regulatory genes screened in 2018 based on AMMI model, coefficient of variation (CV) and average phenotypic value. Dotted line represents value of wild-type. ASV: AMMI stability value.


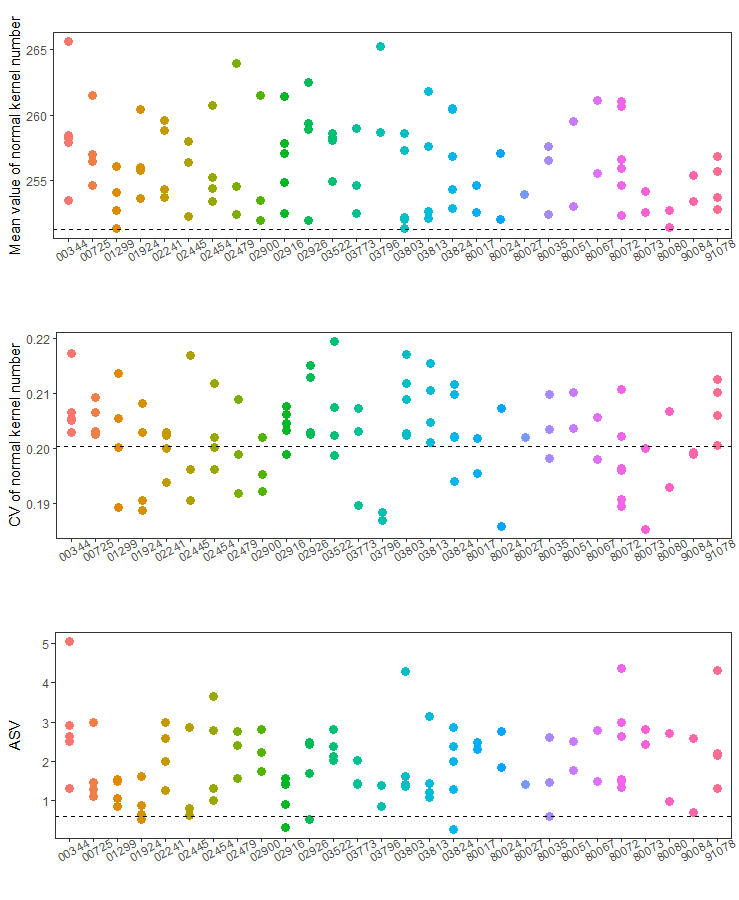


**Fig. S12** Analyzing the performance of 28 candidate regulatory genes screened in 2019 based on AMMI model, coefficient of variation (CV) and average phenotypic value. Dotted line represents value of wild-type. ASV: AMMI stability value.


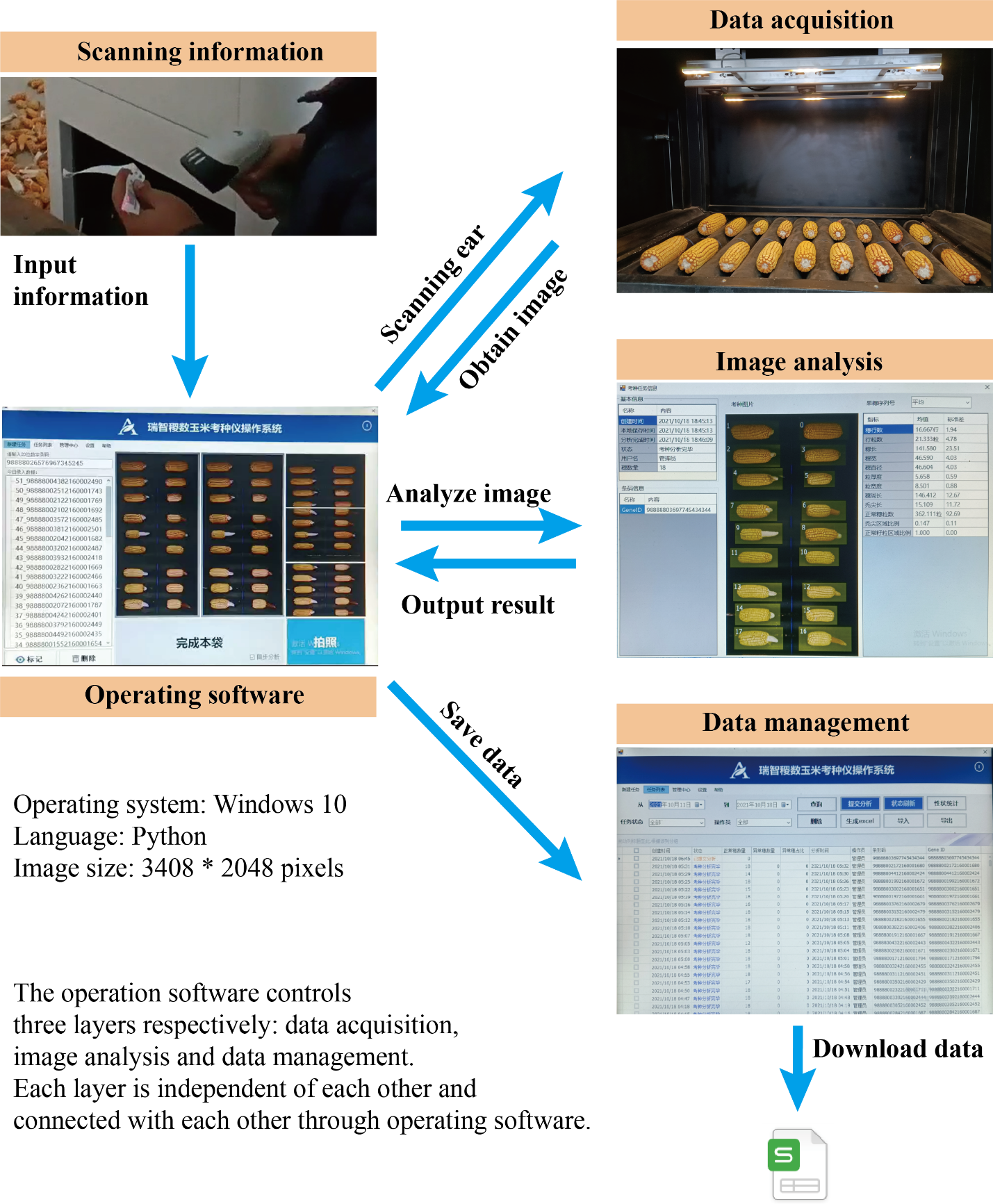


**Fig. S13** Flow chart of relationship between different layers of MAIZTRO.


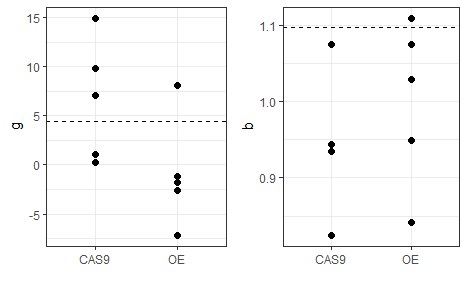


**Fig. S14** Effect of overexpression and knockout of *GRMZM2G077278* on phenotypic plasticity for normal kernel number in 2018.

**Fig. S15** Plot from FWR analysis of genes that performed well and were present in lines planted in both 2018 and 2019 (n = 10 genes).
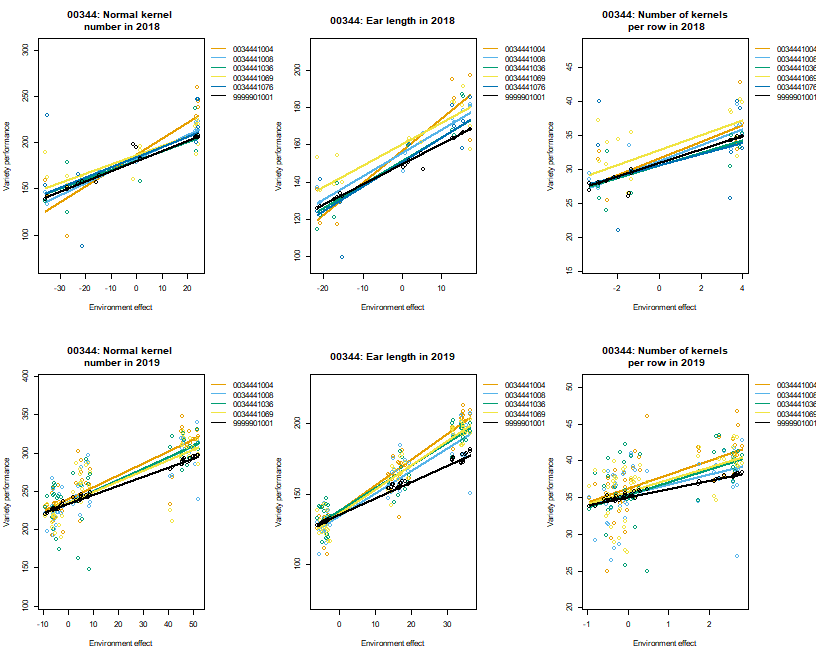

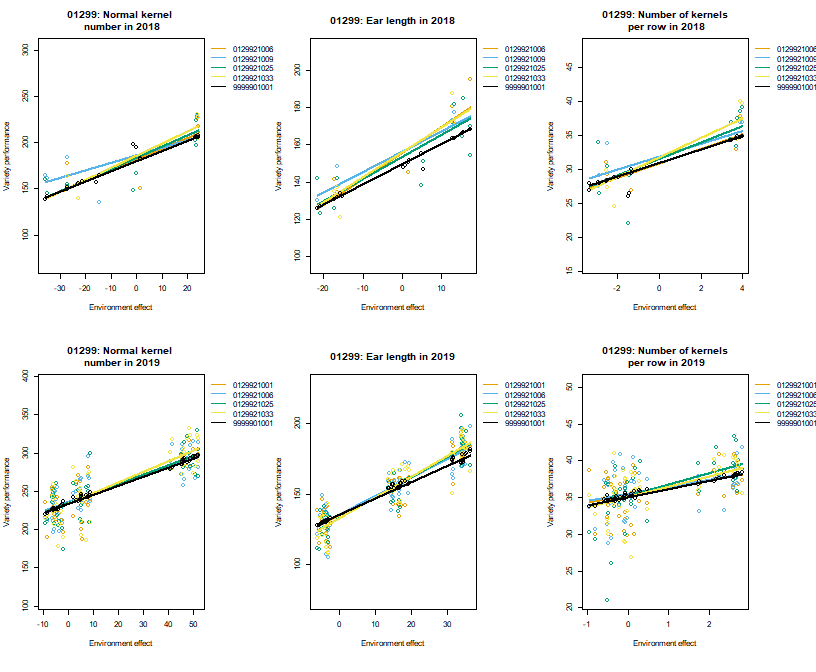

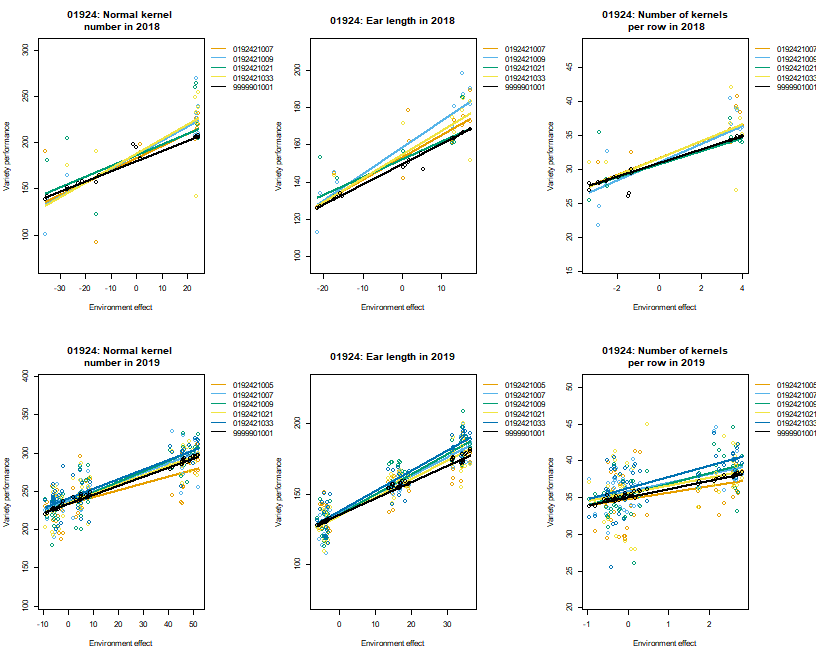

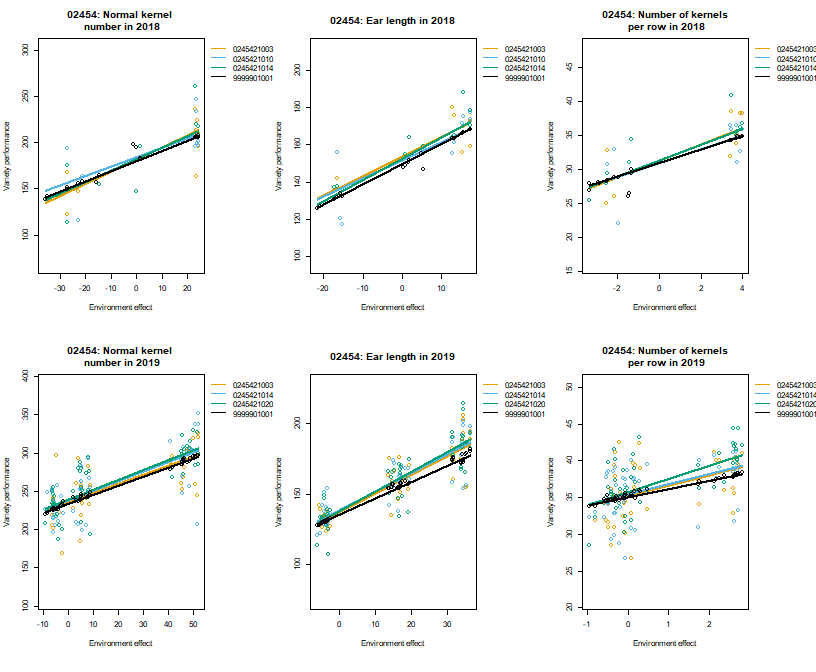

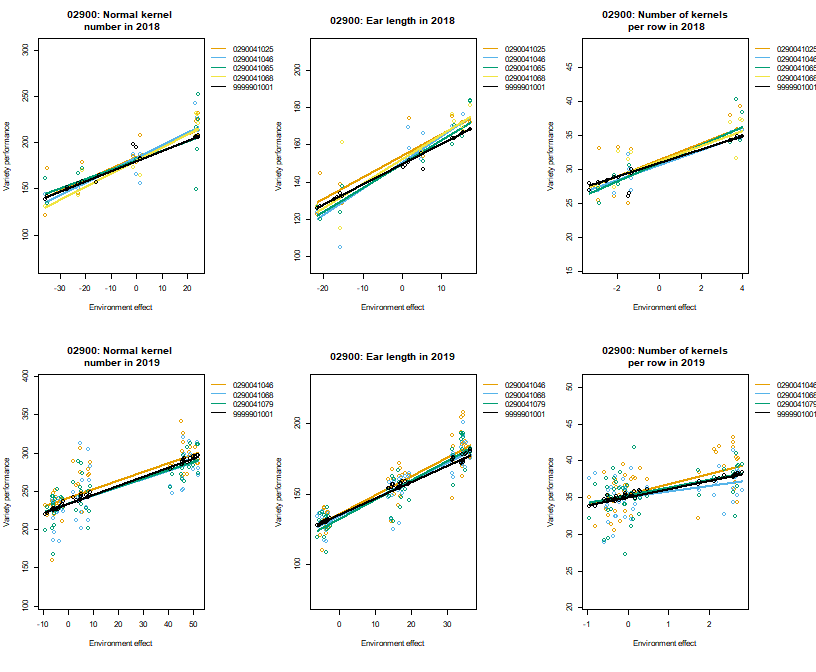

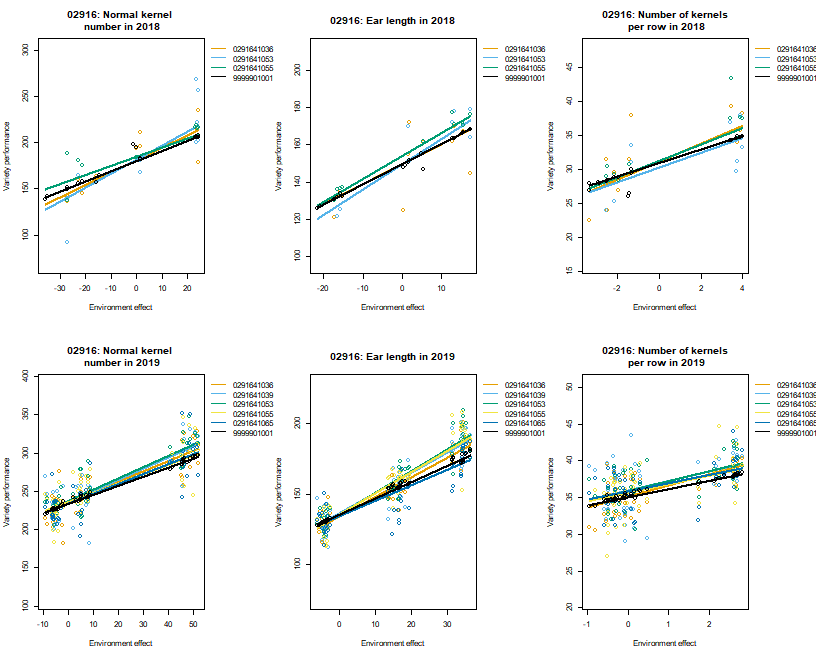

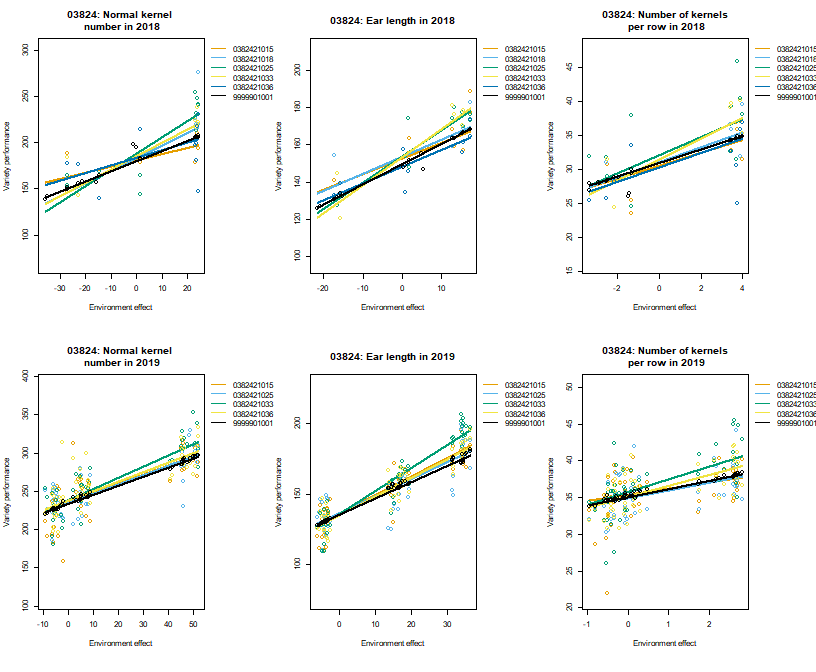

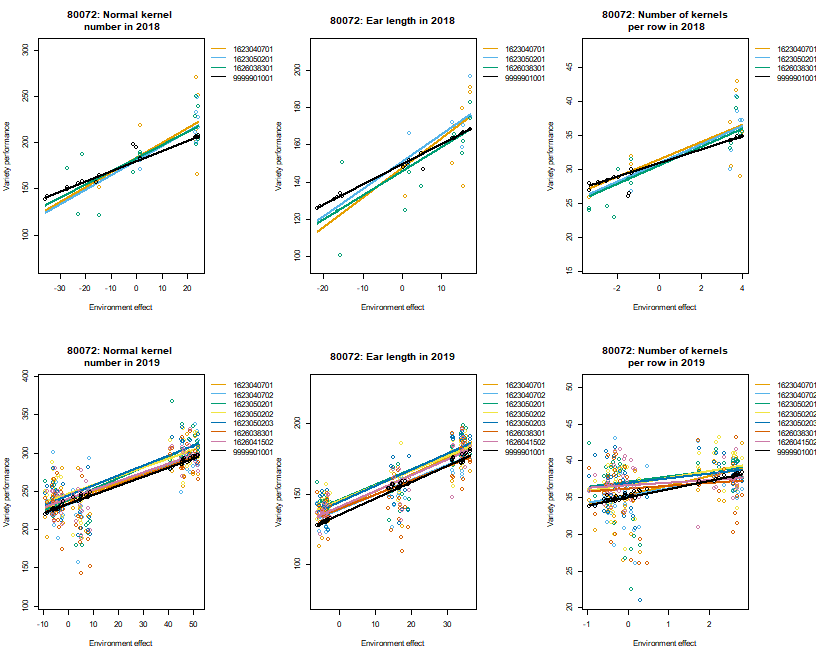

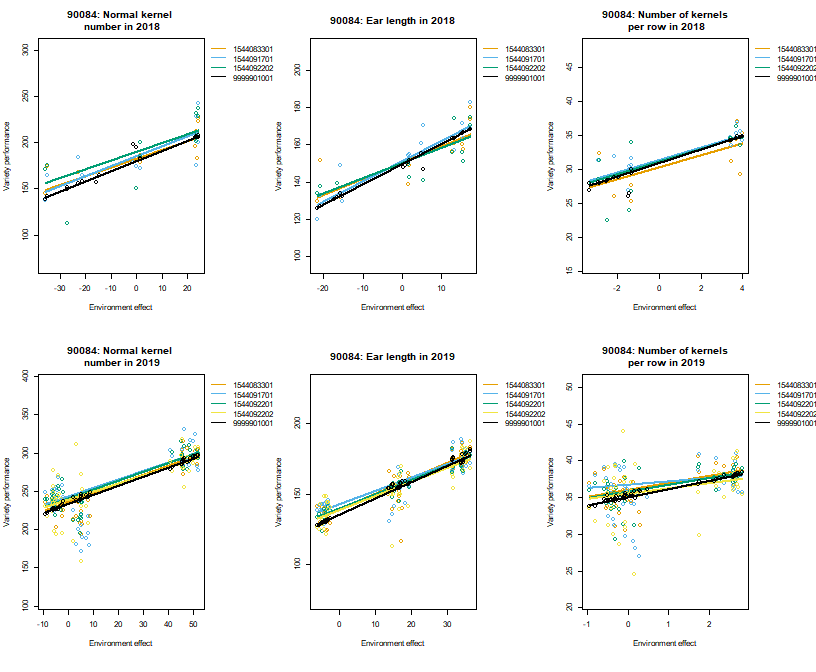

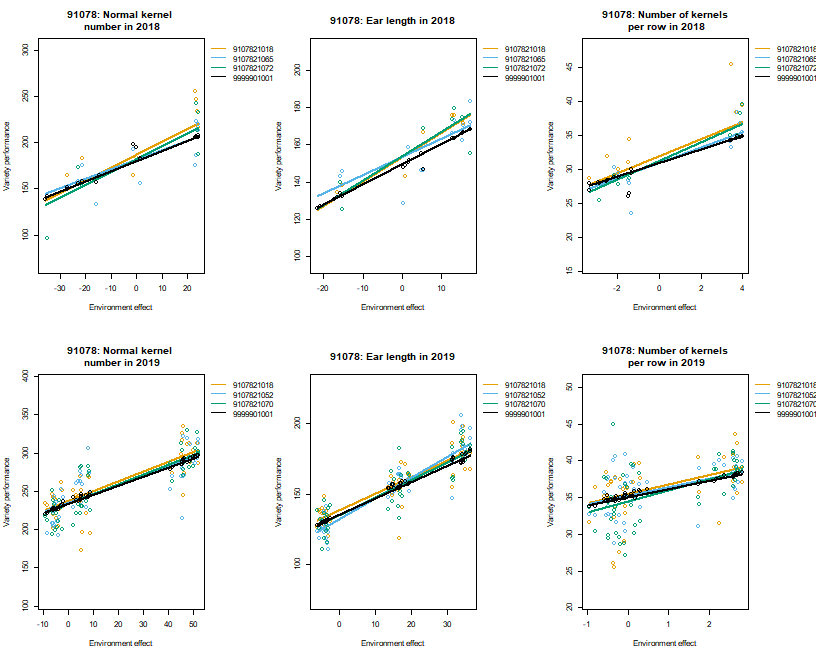


**Fig. S16** Plot from FWR analysis of genes that performed well and were present in lines planted only in 2018 (*n* = 6 genes).


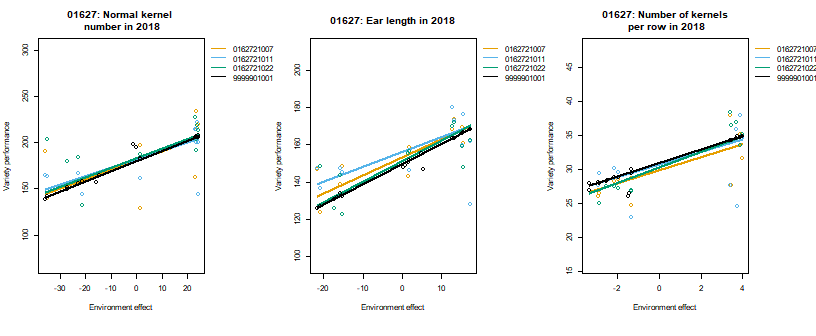

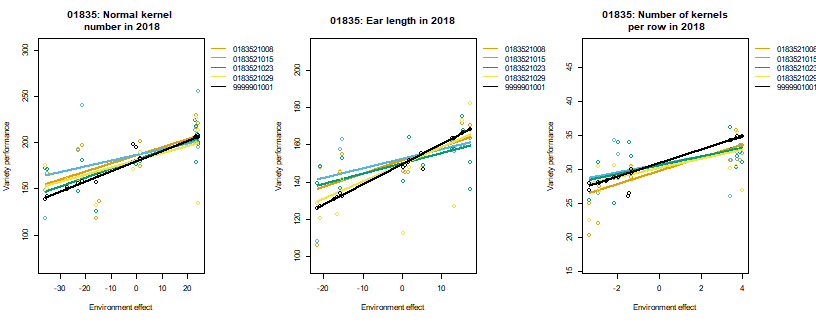

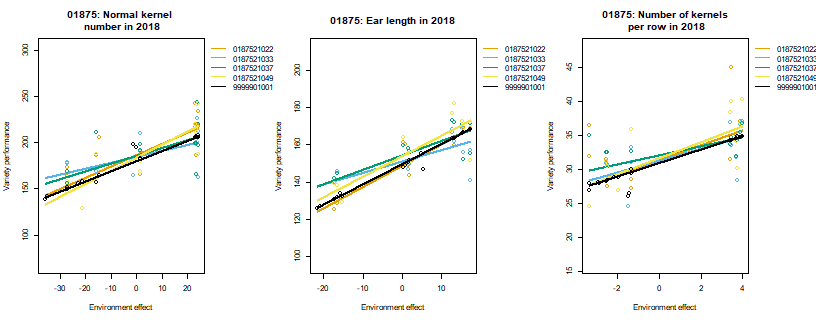

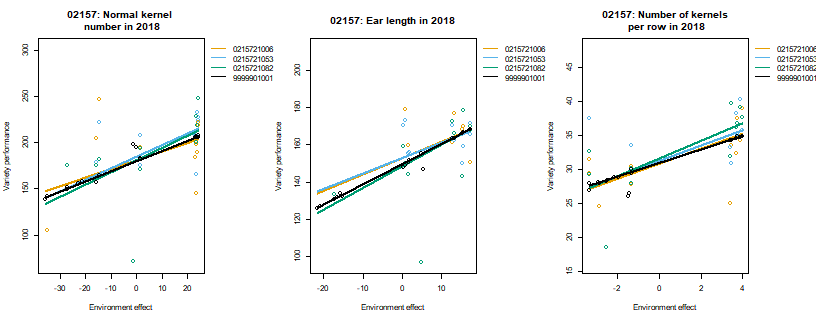

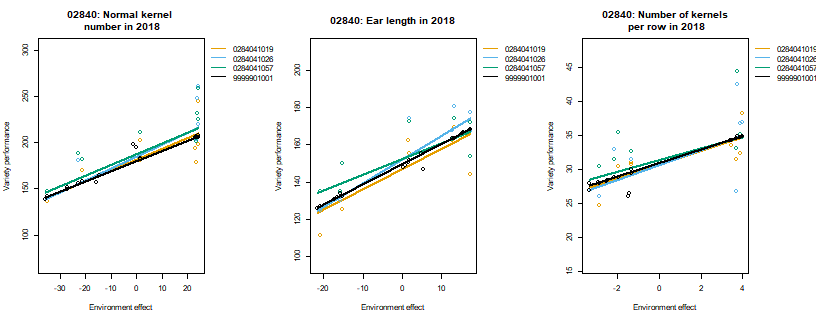

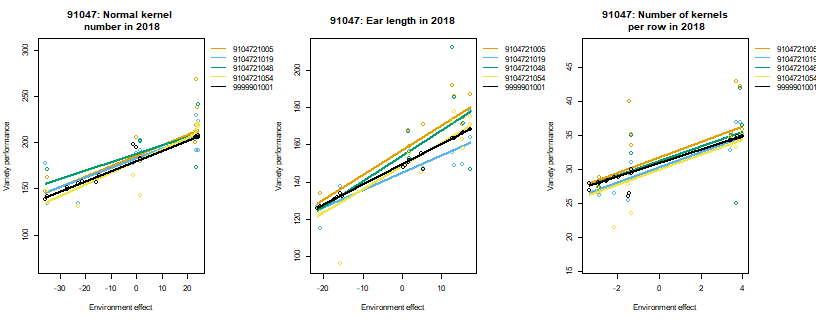


**Fig. S17** Plot from FWR analysis of genes that performed well and were present in lines planted only in 2019 (*n* = 18 genes).


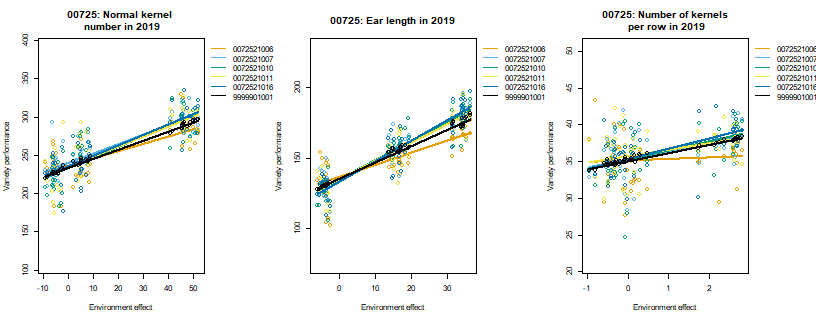

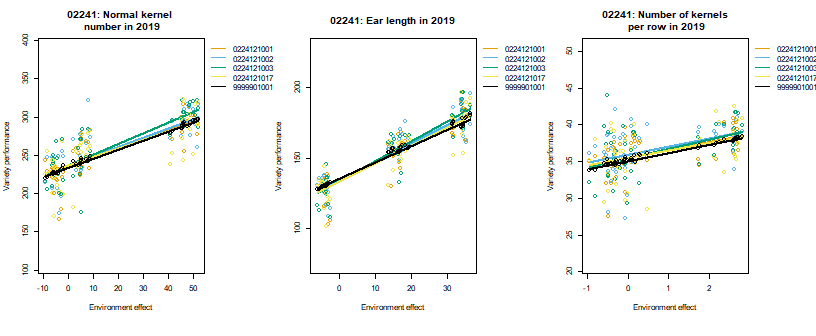

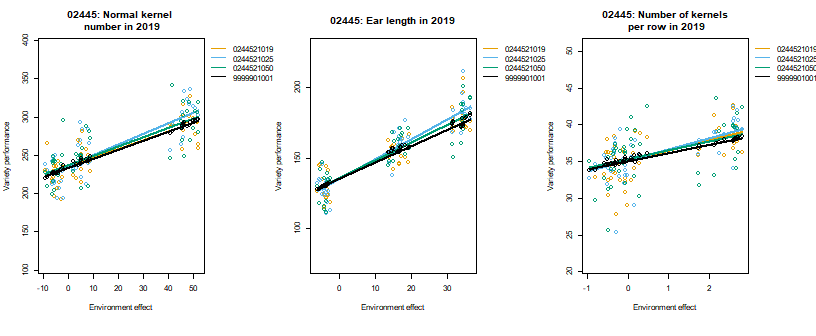

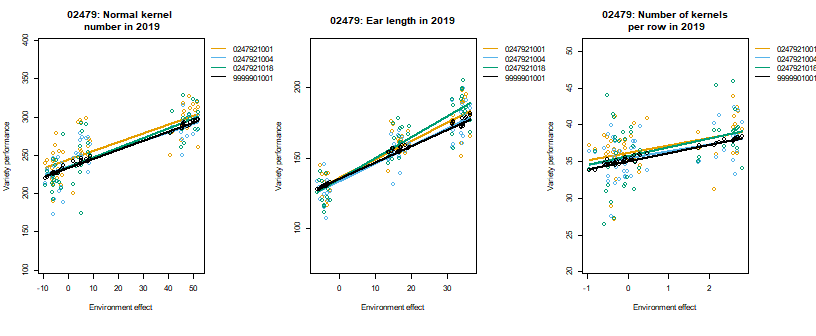

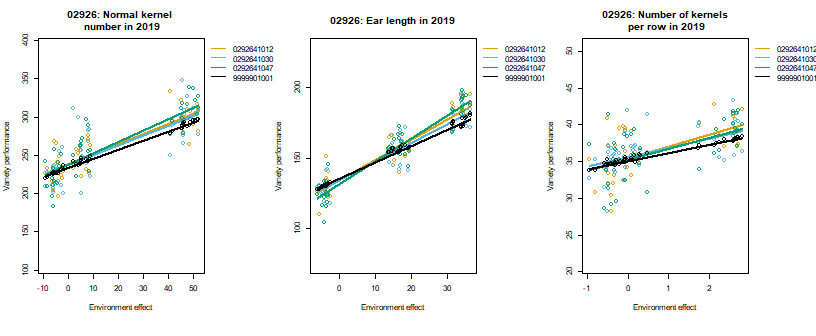

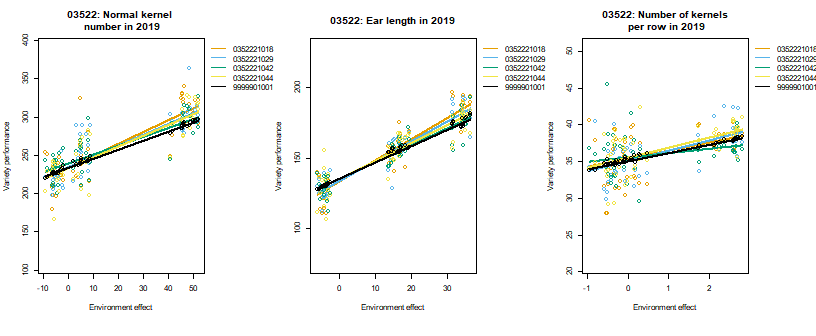

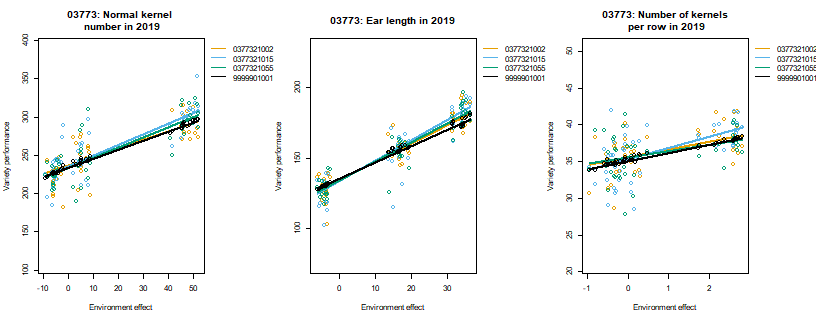

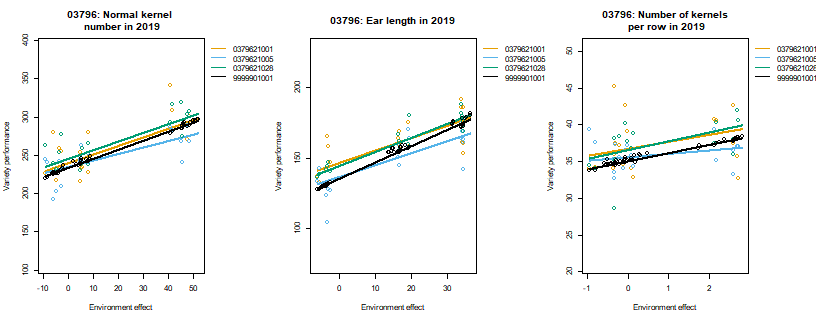

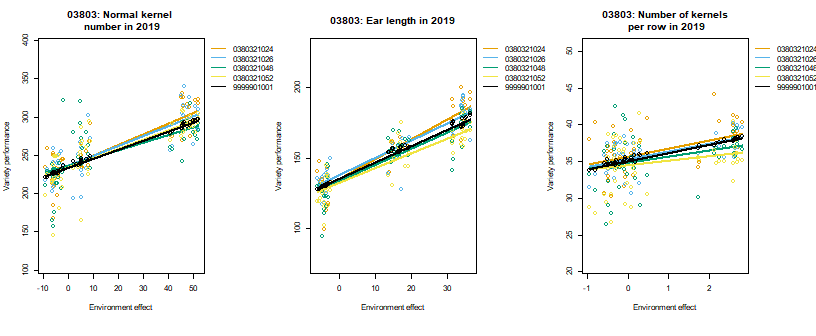

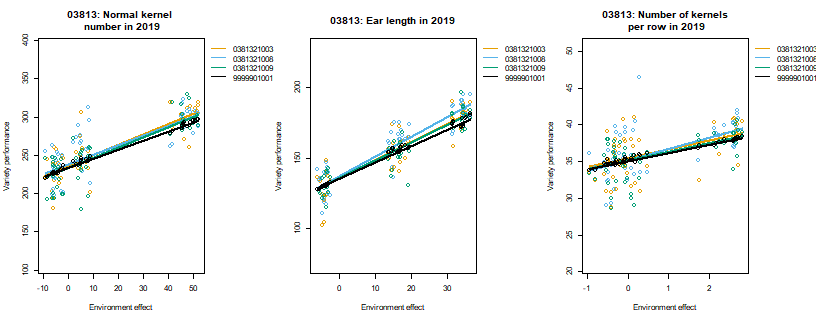

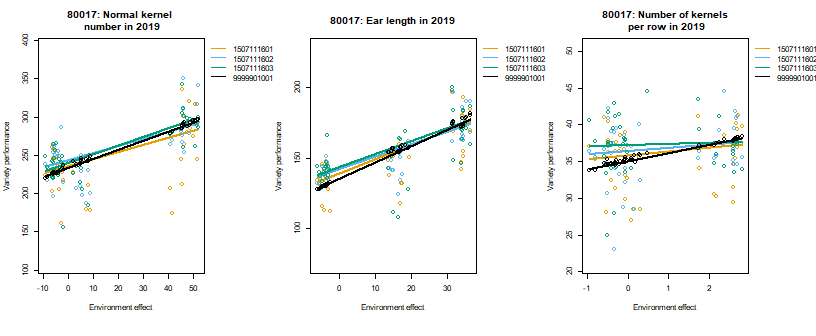

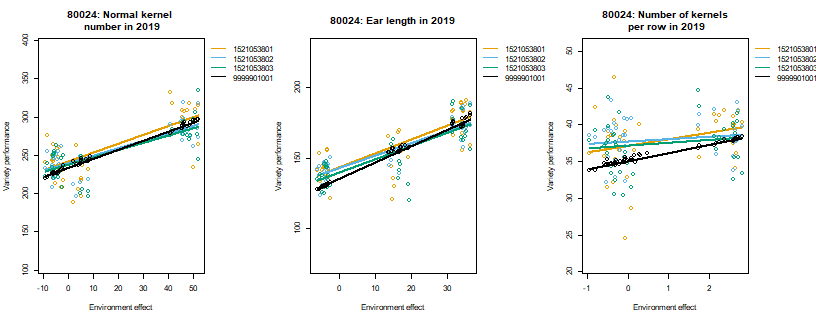

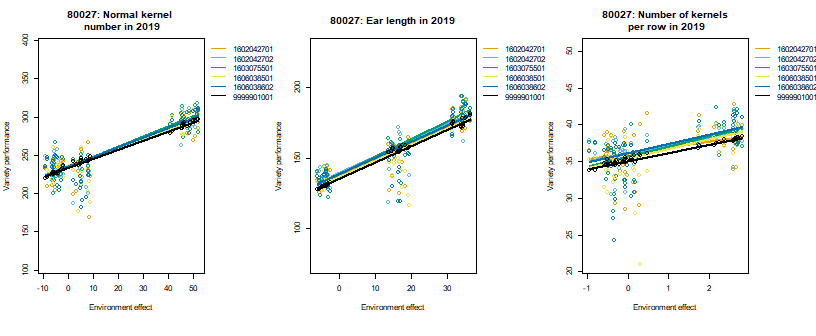

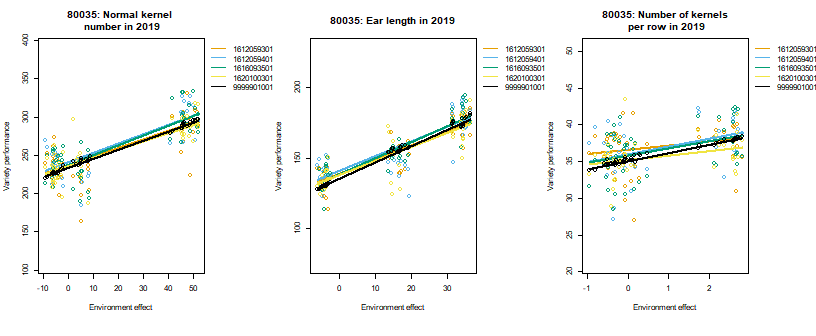

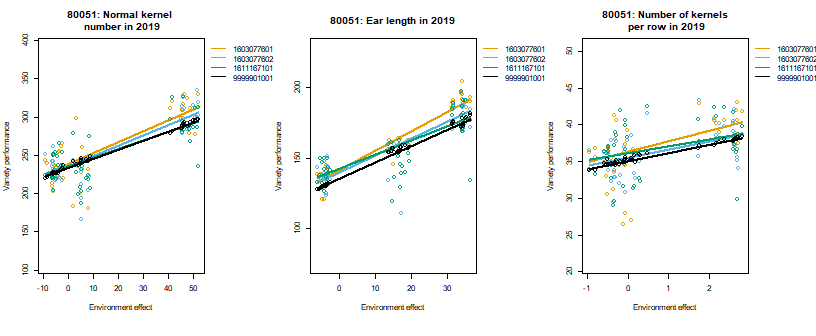

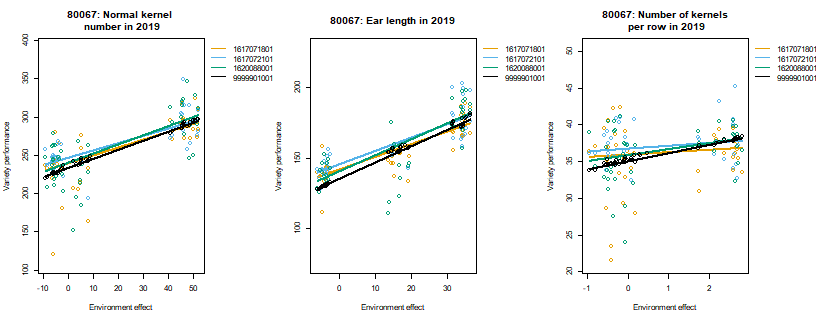

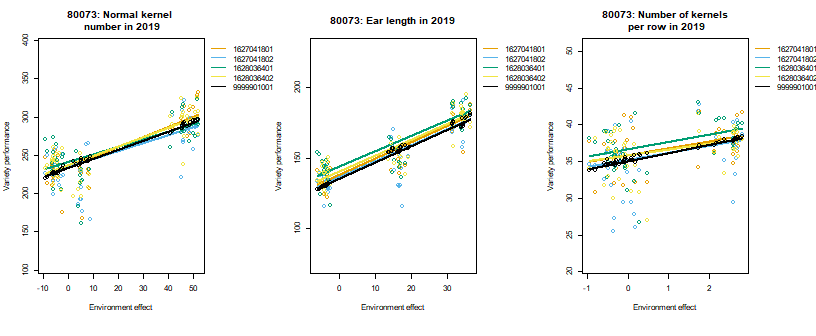

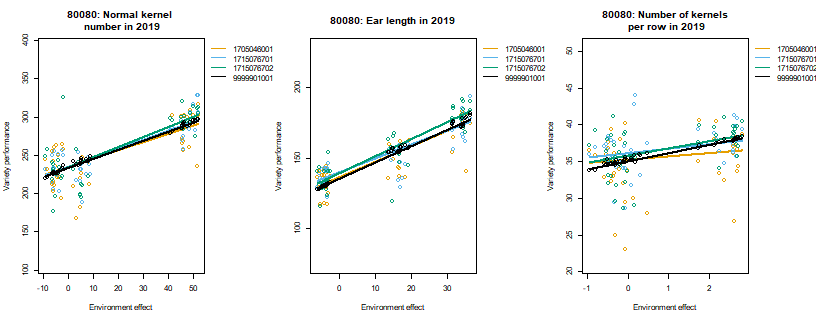

Supplement: Supplementary file 1 — Additional file 1: Figure S1. Phenotypic variation of ears from transgenic inbred lines planted in 2018. Figure S2. Phenotypic variation of ears from transgenic inbred lines planted in 2019. Figure S3. Distribution of the coefficient of variation for transgenic inbred lines planted in 2018. Figure S4. Distribution of the coefficient of variation for transgenic inbred lines planted in 2019. Figure S5. Correlation matrix plot of phenotypic ear characteristics of transgenic inbred lines planted in 2019. Figure S6. Genetic correlations among mean phenotype values, linear plasticity, and nonlinear plasticity for transgenic inbred lines planted in 2018. Figure S7. Genetic correlations among mean phenotype values, linear plasticity, and nonlinear plasticity for transgenic inbred lines planted in 2019. Figure S8. Distribution of genetic values and linear plasticity of phenotypes for transgenic inbred lines planted in 2019. Figure S9. Phenotypic plasticity of transgenic lines planted in 2018. Figure S10. Phenotypic plasticity of transgenic lines planted in 2019. Figure S11. Analyzing the performance of 16 candidate regulatory genes screened in 2018 based on AMMI model, coefficient of variation (CV), and average phenotypic value. Figure S12. Analyzing the performance of 28 candidate regulatory screened in 2019 based on AMMI model, coefficient of variation (CV), and average phenotypic value. Figure S13. Flow chart of relationship between different layers of MAIZTRO. Figure S14. Effect of overexpression and knockout of GRMZM2G077278 on phenotypic plasticity for normal kernel number in 2018. Figure S15. Plot from FWR analysis of genes that performed well and were present in lines planted in both 2018 and 2019 (n = 10 genes). Figure S16. Plot from FWR analysis of genes that performed well and were present in lines planted only in 2018 (n = 6 genes). Figure S17. Plot from FWR analysis of genes that performed well and were present in lines planted only in 2019 (n = 18 genes). [file 13059_2023_2937_MOESM1_ESM.docx]
