# Supplementary material for: Identifying yield-related genes in maize based on ear trait plasticity
Source: Genome Biol. 2023 Apr 25;24:94. doi: 10.1186/s13059-023-02937-6 (PMC10127483; doi:10.1186/s13059-023-02937-6)
Supplement: Supplementary file 3 — Additional file 3. Review history. [file 13059_2023_2937_MOESM3_ESM.docx]

Review History

**First round of review**

**Reviewer 1**

**Were you able to assess all statistics in the manuscript, including the appropriateness of statistical tests used?**

Yes, and I have assessed the statistics in my report.

**Comments to author:**

Measurement of phenotypes is an important part of crop genetic improvement. In recent years, , researchers have made great progress in understanding the molecular and genetic mechanisms of crop improvements by using high-throughput phenotyping techniques. In this manuscript, the authors developed a MAIZTRO system for high-throughput measurement of ear traits with combined machine learning methods, and identified multiple potentially valuable high-yielding genes by phenotyping thousands of maize transgenic lines. This work provides useful resources for ear traits improvement. Here are my questions and suggestions for the authors:

1.By comparing the differences between MAIZRO and manual measurements, authors demonstrate the excellent measurement accuracy of MAIZRO system. It is worth noting that from the perspective of phenotypic distribution, most ears used for phenotyping are plump (most of the proportion of bald tip area is less than 10%, and most proportion of empty area is less than 6%, addition file 1). With the increased frequency of extreme environmental stresses in recent years wichi often results in underfilled ears, the need for environment-adapted breeding also grows. I wondered whether MAIZRO has similar performance with under-full ears (For example, the ear with proportion of empty area value over 20%) as it does with full ears.

2.For kernel number measurement, if only one side of the ear is measured, will it affect the measurement accuracy of kernel number? If not,why?

3.At present, the intelligent seed testing machine can be used to quickly and accurately obtain a large number of crop yield related-traits. What are the advantages of MAIZTRO compared with those seed testing machines?

4.Only the Bayesian FWR method was used to evaluate the interaction between genotype and environment, and calculate the g and b values. Other models or methods could be considered to validate the analysis.

5.There are variations among different maize ears and kernels, e.g their shapes and colors are different. The training dataset of MAIZTRO system was generated with 30,000 scanned images of inbred lines or hybrids of tropical, subtropical, and temperate maize varieties. I suggest that the authors provide the measurement accuracy of ears from different areas in the test dataset.

6.Among the screened candidate genes, can the ear traits of overexpression transgenic plants be verified by the knockout materials of the same gene?

7.Are there any genes, which were used to generate transgenic materials by the authors, reported to affect ear traits previously? If yes, whether the transgenic materials of these genes show ear-related phenotypes in the field?

8.The authors identified 34 genes that could affect grain number, and some of these genes, such as GRMZM2G012891, GRMZM2G368838_T01, and GRMZM2G090435_T01, have been reported to be associated with temperature adaptation. It is known that yield traits have been strongly selected during breeding. I would like to know whether these 34 genes are preferred to be selected, and if not, what are the possible reasons. Meanwhile, I suggest that the authors could perform genetic analysis to one or some selected gene(s) in these 34 genes, and provide pictures for some yield-related traits as well as their related statistic data.

9.Phenotypic plasticity is important in crop genetic improvement. How should we consider its application in breeding? Needs discussion.

**Reviewer 2**

**Were you able to assess all statistics in the manuscript, including the appropriateness of statistical tests used?**

Yes, and I have assessed the statistics in my report.

**Comments to author:**

The paper titled "Discovering yield-related genes in maize based on ear trait plasticity" introduces an ear imaging platform and subsequently uses the platform to discover genes involved in plasticity.

Factually, few of the following suggestions change the conclusions of the paper: High-throughput image measurement of transgenic corn ears uncovered genes potentially involved in yield plasticity.

Here is a list of suggestions:

The paper is two papers in one. Have the author's considered marginalizing the imaging solution and magnifying the genes? If the imaging is going to stay then it seems important to rework the justification. For example:

a: Smartgain was built to handle images on a homogenous background. The authors are looking to capture kernels on the ear. Saying that Smartgrain is not suited for the task because of its simplistic modeling misses the point. Smartgrain is not suited for the task due to the complexity of measuring kernels on the ear. Potentially, the only part of smart grain which does translate to the view of the kernels on the ear is the simplistic modeling of the kernel cap as an ovalish type of shape.

b: The authors cite Miller et al. [26] and suggest that a "multiple-algorithms method applied to plant colors and edges" somehow handles diversity. This sentence is ambiguous and seems to suggest that the three methods presented in [26] are comparable to the method presented in this paper. As with smart grain, this is not a valid comparison. The authors should either re-read the papers, highlight the specific comparisons which are lacking or consider a different path for justification.

c: The authors suggest [26] is limited by illumination conditions and image quality. While true, the statement is too broad to add value to the paper. Without a clear definition of illumination and quality, this path for justification will always suggest that image software is lacking.

The authors suggest that the method in Warman et al. [27] is not suitable for field applications? Does this refer to the software? The imaging platform? Again, the authors are looking to justify their software, they need to be more specific.

Consider one of the following: marginalize the software and imaging components of the paper which will reduce their need for justification or spend the time to highlight the novel aspects of MAIZTRO which are not available elsewhere and are important to the discoveries in the paper. For example: a) MAIZTRO produces a kernel count where [26] does not b) MAIZTRO highlights different regions of the ear where others [26] do not. Highlighting where the community needs help is a fine way to justify the creation of novel software. I encourage the authors to be more specific in this part of the paper.

The authors suggest that an integrated software and hardware platform is needed. Why? While this may seem trivial at first thought, careful consideration reveals integration between the data acquisition and analysis should be carefully considered. If integration is deemed the best approach, then providing the readers with an understanding of how the data acquisition software integrates with the downstream analysis software is important. For example: Did the authors choose a "modular" integration between these layers so that problems in the image analysis would not affect the data acquisition? Can the analysis layer be shared with the public or is the software system not modular in nature? Other simple details are missing from the text, such as: What language is the analysis written in? What operating system is the system running? What resolution are the images?

The paper suggests that "in field" imaging is lacking however the pictures of the imaging device are shown as being used indoors. Please specify what "in field" means, why it is important, why they believe it is necessary and how it is different from "out of field".

The authors present a table with metrics for the algorithms however it is unclear with respect to the training, testing or validation. What percent of the data was held out during training? Was attention given to performing structured hold-outs by genotype or variety? For example, what happens if the authors leave out the tropical variety and only train on the rest? Do their metrics change? What if they leave out dent corn? Overall, it is the authors responsibility to demonstrate they have not over fit their models. Currently, the paper is lacking in this respect. Please provide more information to ensure the reader that the model is not overfitting the data.

The data on github seems well organized however understanding the function of different scripts, what the structure of the data is and how to interact with the data is missing. Please fill out the readme file. This should include detailed information about how to use the data. A well documented large dataset alone has value for the community and will enable if if time is taken to document how to load the data.

I repeat, few of the above suggestions change the conclusions of the paper: high-throughput image measurement of transgenic corn ears uncovered genes potentially involved in yield plasticity. The results presented here are important however details and justification could use attention.

Below is a list of necessary fixes:

a: Ensure the reader that the extraction method is not overfitting the data. Provide structured holdouts by randomizing varieties and holding out varieties.

b: Include more details, for example the resolution of the images.

c: Provide a better understanding of the term "in-field".

Please remove the word "urgent" on line 97.

**Authors Response**

**Point-by-point responses to the reviewers’ comments:**

**Reviewer 1**

Measurement of phenotypes is an important part of crop genetic improvement. In recent years, researchers have made great progress in understanding the molecular and genetic mechanisms of crop improvements by using high-throughput phenotyping techniques. In this manuscript, the authors developed a MAIZTRO system for high-throughput measurement of ear traits with combined machine learning methods, and identified multiple potentially valuable high-yielding genes by phenotyping thousands of maize transgenic lines. This work provides useful resources for ear traits improvement. Here are my questions and suggestions for the authors:

1. By comparing the differences between MAIZRO and manual measurements, authors demonstrate the excellent measurement accuracy of MAIZRO system. It is worth noting that from the perspective of phenotypic distribution, most ears used for phenotyping are plump (most of the proportion of bald tip area is less than 10%, and most proportion of empty area is less than 6%, addition file 1). With the increased frequency of extreme environmental stresses in recent years wichi often results in underfilled ears, the need for environment-adapted breeding also grows. I wondered whether MAIZRO has similar performance with under-full ears (For example, the ear with proportion of empty area value over 20%) as it does with full ears. ***Reply:*** *This is an important question, and thus we have performed an experiment to test MAIZTRO’s prediction performance on underfilled ears. We chose 150 ears with different levels of fullness (Figure R1), measured phenotypic various ear traits using both MAIZTRO and manual methods, and then calculated the prediction accuracy of MAIZTRO for these traits (Table R1). The accuracy for five of the six traits was over 85%, with the highest accuracy for ear width (90%) and the lowest for bald tip length (54.9%). Thus, MAIZTRO’s accuracy on underfilled ears is indeed low compared to normal ears. This may be attributable in part to the small number of such ears and their low proportion in the training data, which could reduce the model’s prediction accuracy for such ears. Moreover, it is generally difficult to measure phenotypic traits of underfilled ears using any method. For instance, the irregular shape of the ear will affect ear length and width, and the irregular kernel distribution will affect the number of rows per ears. In contrast, ear width is relatively easy to measure, so its accuracy is expected to be high. Therefore, measurements and predictions of underfilled ears are inherently limited by their morphology.*

*That said, this should not have a significant impact on the application of MAIZTRO for several reasons. First, underfilled ears would be the first to be eliminated from any breeding efforts. Materials like this are easily identifiable and would not even be measured, since they are clearly not ideal. Second, although the measurement accuracy of MAIZTRO is relatively low compared with that of normal ears, most of the indicators are still above 85%, which will not affect one’s judgment on the phenotypic traits of the two types of ears. Third, the relatively low percentage of such material would not affect the contribution of MAIZTRO to the overall work because of its clear advantages in speed and number of indicators. Finally, it should be noted that users would still have difficulty measuring such ears even if manual measurements were used.*

**Table R1** Accuracy of MAIZTRO in underfilled ears.

| Trait | Accuracy (%) |
| --- | --- |
| Bald tip length | 54.9 |
| Ear length | 87.6 |
| Ear width | 90.0 |
| Normal kernel number | 81.3 |
| Number of kernels per row | 85.8 |
| Number of rows per ear | 88.3 |

**Figure R1** Sample images of underfilled ears.

2. For kernel number measurement, if only one side of the ear is measured, will it affect the measurement accuracy of kernel number? If not,why?

***Reply:*** *This point has been addressed in the revised manuscript (L136-137). During the measurement process, each ear is photographed twice, once from each side. The roller-shaped base for measuring the ears makes it simple not only to keep the ears steady, but also to turn them so that both sides can be photographed. This enables us to accurately measure the normal kernel number of each ear. In the manuscript, this is shown in Fig. 1F and 1H. Here, we provide a more obvious image (Figure R2).*

**Figure R2** Device for placing ears in MAIZTRO.

3. At present, the intelligent seed testing machine can be used to quickly and accurately obtain a large number of crop yield related-traits. What are the advantages of MAIZTRO compared with those seed testing machines?

***Reply:*** *MAIZTRO has some obvious advantages over other ear measurement systems. First, after years of development and adjustment, MAIZTRO has realized the integration of software and hardware, forming a complete ear measurement workflow and a mature product. Articles describing some maize ear measurement systems are limited because they are still in the process of developing algorithms and simple devices (Makanza et al., 2018; Warman et al., 2021). Second, the operation of MAIZTRO is simple and automated. Plot information is quickly obtained using a hand-held scanner, and ear scanning, analysis, and data download are performed through an easy-to-use interface. These features allow MAIZTRO to be used by ordinary staff after simple training. In contrast, some other devices require complex system settings that may require more specialized skills, e.g., computer programming (Miller et al., 2017). Third, MAIZTRO has high measurement efficiency, with the ability to measure 18 ears at a time, about 700 times a day, for a total of about 12,600 ears a day. Some ear measurement systems can only measure one ear at a time (Warman et al., 2021). Fourth, MAIZTRO can obtain more ear traits. In addition to analyzing ear length and width and kernel number (Miller et al., 2017; Warman et al., 2021), MAIZTRO can analyze regional proportions and quantities of different kernel types that are often of interest to breeders. A total of 15 ear phenotypes can be analyzed: bald tip length, ear circumference, ear diameter, ear length, ear width, kernel thickness, kernel width, normal kernel number, shrunken kernel number, number of kernels per row, number of rows per ear, proportion of bald tip area, proportion of empty area, and proportion of normal seed area. Fifth, MAIZTRO operates as a relatively closed and flexible system. It is highly suitable for use in the field because the closed setting prevents interference from environmental factors. Compared with other open structures, MAIZTRO is more suitable for changing field conditions (Makanza et al., 2018; Warman et al., 2021). The mobile design of the platform is very convenient for use in the field. In the future, we hope to include a built-in power supply, which will further improve its ease of use in the field.*

*We have addressed these advantages in the Discussion section at L276-287.*

4. Only the Bayesian FWR method was used to evaluate the interaction between genotype and environment, and calculate the g and b values. Other models or methods could be considered to validate the analysis.

***Reply:*** *Yes, other models or approaches may be considered for the validation of the analysis. The AMMI (Additive Main Effects and Multiplicative Interaction) model is another essential technique for analyzing gene-environment interactions (Crossa, 1990). Here, we utilize the AMMI model to assess the stability of normal kernel number of different events, the coefficient of variation to compute the degree of dispersion of traits, and the mean value to represent the* *value of the phenotypic trait in both years (Figures R3 & R4). The dominant genes screened by us had obvious advantages in normal kernel number compared with wild-type materials and had a significantly higher normal kernel number. The AMMI stability value (ASV) was higher than that of wild-type materials, and the coefficient of variation was distributed around wild-type materials. This analysis confirms that the genes we screened have obvious advantages in relation to increasing the normal kernel number (L313-317).*

*The Bayesian FWR method we used in this paper is a better method for gene-environment interaction analysis. First, it can show not only the stability characteristics of different materials but also the trend of phenotypic characteristics changing with environmental effects. This is of great significance for analyzing the trends of phenotypic changes with environmental changes. Second, this method not only outputs stability features (b), but also outputs average phenotypic features (g). This average phenotypic feature is different from the average value calculated by other methods, but makes a more stable inference based on the model. Third, this method infers model FWR parameters based on a Bayesian model, which can obtain more stable model parameters (Lian and de los Campos, 2016). In addition, FWR can adapt to the analysis of large datasets because it is developed in C++ (Lian and de los Campos, 2016). In contrast, other methods and models are mainly built on R, which is not suitable for analysis of large amounts of data.*

*Several significant studies have currently used this method to estimate the phenotypic plasticity of various materials (maize GWAS materials) (Gage et al., 2017; Kusmec et al., 2017; Liu et al., 2020).*

**Fig. R3** Analysis of performance of 16 genes screened in 2018 based on the AMMI model, coefficient of variation (CV), and average phenotypic value. Dotted line represents phenotype of wild type materials. ASV: AMMI stability value.

**Fig. R4** Analysis of performance of 28 genes screened in 2019 as described for Fig. R3.

5. There are variations among different maize ears and kernels, e.g their shapes and colors are different. The training dataset of MAIZTRO system was generated with 30,000 scanned images of inbred lines or hybrids of tropical, subtropical, and temperate maize varieties. I suggest that the authors provide the measurement accuracy of ears from different areas in the test dataset.
***Reply:*** *The ears used in our training model did indeed come from a variety of regions and genetic backgrounds. Unfortunately, we neglected to record the source information during collection, making it impossible to verify the accuracy of the model based on source information.*

*In order to evaluate the prediction performance of MAIZTRO on different types of ears, we divided ears into seven groups according to morphological characteristics as defined in Table R2, and then calculated the prediction accuracy of MAIZTRO in the different groups. The results indicate that MAIZTRO has high precision (Most are greater than 95%) for different types of ears except bald tip length. The irregular shape of bald tip makes it difficult to determine its length.*

**Table R2** Measurement accuracy of ears with different morphological characteristics.

| Index | G1 | G2 | G3 | G4 | G5 | G6 | G7 |
| --- | --- | --- | --- | --- | --- | --- | --- |
| Bald tip length | 62.2 | 72.5 | 71.1 | 76.2 | 85.0 | 72.9 | 72.9 |
| Ear length | 98.3 | 97.9 | 97.4 | 97.6 | 98.1 | 98.4 | 96.6 |
| Ear width | 98.2 | 98.0 | 97.9 | 98.1 | 98.2 | 98.0 | 98.7 |
| Normal kernel number | 93.3 | 92.9 | 92.7 | 95.8 | 94.4 | 94.7 | 93.7 |
| Number of kernels per row | 92.8 | 89.9 | 90.2 | 92.1 | 91.3 | 89.8 | 93.6 |
| Number of rows per ear | 96.7 | 96.4 | 97.4 | 97.3 | 97.5 | 97.8 | 98.0 |

G1: Ears with complex colors; G2: damaged ears; G3: ears with irregular kernel distribution; G4: ears with spirally distributed kernels; G5: long bald ears; G6: normal ears; G7: wide ears.

6. Among the screened candidate genes, can the ear traits of overexpression transgenic plants be verified by the knockout materials of the same gene?
***Reply:*** *Yes, the function of specific genes can be better explained by the opposite gene treatment, but we did not simultaneously overexpress and knockdown these 34 candidate genes separately. This would have required significant additional costs and time (about 3 years from material creation to seed production to field phenotype observation), and thus we were unable to perform complete validation of all transgenic material in a timely fashion. In our daily work, our aim is to find new materials, not to analyze gene function in detail, so we will only verify some of the genes of interest. Fortunately, one of our 34 candidate genes was subjected to both overexpression and knockout treatments.* *Knockout of GRMZM2G077278 increased the normal kernel number, whereas overexpression significantly reduced the normal kernel number. Thus, this gene serves as an example of the potential application of this high-throughput phenotype platform and the transgenic materials in mining of potential dominant genes. This has been added to L330-332 of the manuscript, and the results have been added to the Supplementary Information as Additional file 1: Figure S14.*

*We do not believe that the lack of validation for all 34 candidate genes detracts from the subject of this paper. In this paper, we have demonstrated a more efficient way to identify maize yield-related genes in transgenic maize populations grown in different environments using novel tools with advantageous features.*

**Fig. S14** Effect of overexpression and knockout of *GRMZM2G077278* on phenotypic plasticity for normal kernel number in 2018.

7. Are there any genes, which were used to generate transgenic materials by the authors, reported to affect ear traits previously? If yes, whether the transgenic materials of these genes show ear-related phenotypes in the field?
***Reply:*** *Of the 34 genes screened by us, 8 were reported previously, but none had previously known maize ear phenotypes. We have added some information on these genes in the Discussion section at L323-327. GRMZM2G333183 is a key gene that responds to salt stress (Ma et al., 2021; Jia et al., 2022) is also related to P stress (Du et al., 2016). GRMZM2G004483 plays a role in biological processes related to flowering time, such as reproduction, flower development, and post-embryonic development (Song et al., 2017; Zhang et al., 2018). It was shown to play an important role in regulating maize photoperiod flowering and helping maize adapt to higher latitudes (Huang et al., 2017). GRMZM2G178795 has been associated with photoperiod sensitivity (Yang et al., 2013). Wei et al. (2018) identified GRMZM2G093195 as a maize dwarf gene (D3). There is evidence that GRMZM2G099425 is a calcium-dependent protein kinase and may be involved in calcium signaling, which is a major mechanism involved in* *maize heat tolerance (Frey, 2015). It has been suggested that GRMZM2G134708 is related to cold stress (Avila, 2016; Avila et al., 2018). GRMZM2G328785 is highly expressed in most maize organs and stages of development except for the endosperm and embryo, and may play an important role in maize development prior to the formation of embryo models (Wei et al., 2014). GRMZM2G077278 has been identified as a biomass accumulator in maize (Sun et al., 2021) that contributes to energy balance and distribution in plants (Wang et al., 2021).*

*Although none of these genes are directly related to ear phenotypes, they are involved in processes of environmental stress adaptation and photoperiod adaptation in maize, which are* *closely related to growth and development.*

8. The authors identified 34 genes that could affect grain number, and some of these genes, such as GRMZM2G012891, GRMZM2G368838_T01, and GRMZM2G090435_T01, have been reported to be associated with temperature adaptation. It is known that yield traits have been strongly selected during breeding. I would like to know whether these 34 genes are preferred to be selected, and if not, what are the possible reasons. Meanwhile, I suggest that the authors could perform genetic analysis to one or some selected gene(s) in these 34 genes, and provide pictures for some yield-related traits as well as their related statistic data.
***Reply:*** *As you mentioned, yield traits have been strictly selected in the breeding process. In order to analyze whether our 34 candidate genes were selected in this selection process, we compared them with genes selected in the process of maize domestication and improvement. Hufford et al. (2012) reported a comprehensive evaluation of the evolution of modern maize based on the whole-genome rearrangement of 75 wild and local varieties and improved maize lines, and analyzed the genes involved in the process of domestication and improvement. We found that 4 of our 34 genes (GRMZM2G178795, GRMZM2G123387, GRMZM2G111906, and GRMZM2G126505) were among the selected genes during maize domestication and improvement. Although not all 34 candidate genes were reported by Hufford et al. (2012), it can be concluded that our analytical approach is capable of identifying genes that were retained by maize during domestication and selection. This information has been added to L327-329.*

*Kusmec et al. (2017) report the results of genome-wide association analyses of multiple phenotypes and two measures of phenotypic plasticity in a maize nested association mapping (US-NAM) population grown in multiple environments and genotyped with ~2.5 million single-nucleotide polymorphisms. They found potential candidate genes regulating the plasticity of multiple growth phenotypes and ear phenotypes in maize. We referred to their results and found that two genes in their GWAS were consistent with genes we screened from materials in 2019. Two genes, GRMZM2G414460 and GRMZM2G347361, which have been identified from significant SNPs in the mean phenotype of growing degree days (GDD) to silk and cob diameter (Kusmec et al., 2017). This confirmed the potential role of our 34 genes in regulating phenotypic plasticity from another perspective.*

9. Phenotypic plasticity is important in crop genetic improvement. How should we consider its application in breeding? Needs discussion.

***Reply:*** *Phenotypic plasticity is defined as the phenotypic variation of a trait when an organism is exposed to different environments, which can reflect the stability of specific phenotypes of materials. We can use this method to evaluate the yield stability of different materials under different environments. In breeding, we hope to obtain a material with increased yield compared* *with the reference material in both high- and low-yield environments in order to improve yield stability. Our method can identify genes and gene functions that contribute to the yield increases under both high- and low-yield conditions. This is conducive to enriching germplasm materials, providing help for breeding, and discovering materials or genes best suited for different regions. We have addressed this at L345-353 in the Discussion section.*

**Reviewer 2**

The paper titled "Discovering yield-related genes in maize based on ear trait plasticity" introduces an ear imaging platform and subsequently uses the platform to discover genes involved in plasticity.

Factually, few of the following suggestions change the conclusions of the paper: High-throughput image measurement of transgenic corn ears uncovered genes potentially involved in yield plasticity.

*Reply: We are grateful for the many valuable comments made by the reviewer, which helped improve the article. We respond to each comment below.*

Here is a list of suggestions:

10. The paper is two papers in one. Have the author's considered marginalizing the imaging solution and magnifying the genes? If the imaging is going to stay then it seems important to rework the justification. For example:

***Reply:*** *Indeed, the paper does consist of two parts: one highlighting a newly developed maize ear phenotypic measurement system (MAIZTRO) and one highlighting its use to uncover genes that may affect the plasticity of maize ear yield based on transgenic maize populations. Combining these parts enable us to state that the goal is to develop novel tools and traits (in response to agricultural needs) that will lead to novel discoveries through the use of automation and artificial intelligence. We believe these two components are necessary and complementary, and we have attempted to balance them to achieve our goals.*

10a. Smartgain was built to handle images on a homogenous background. The authors are looking to capture kernels on the ear. Saying that Smartgrain is not suited for the task because of its simplistic modeling misses the point. Smartgrain is not suited for the task due to the complexity of measuring kernels on the ear. Potentially, the only part of smart grain which does translate to the view of the kernels on the ear is the simplistic modeling of the kernel cap as an ovalish type of shape.
***Reply:*** *As you point out, Smartgain mainly handles images on a homogenous background. It uses traditional image processing technology to acquire* *kernel objects in the image, which is difficult to do on a maize ear. We have revised this part of the description in L89-93.*

10b. The authors cite Miller et al. [26] and suggest that a "multiple-algorithms method applied to plant colors and edges" somehow handles diversity. This sentence is ambiguous and seems to suggest that the three methods presented in [26] are comparable to the method presented in this paper. As with smart grain, this is not a valid comparison. The authors should either re-read the papers, highlight the specific comparisons which are lacking or consider a different path for justification.

***Reply:*** *This diversity has been addressed* *by combining traditional image processing with machine learning technology (e.g., fast Fourier transform) [26].* *However, a method based on gray signal periodicity to analyze kernel characteristics is limited by the object's color and its distribution. The calculation of kernel numbers depends on the counting of separate kernels, which inevitably increases the workload. Additionally, the configuration of the system is relatively complex and has relatively high requirements for user skills. This is addressed in L93-99.*

10c. The authors suggest [26] is limited by illumination conditions and image quality. While true, the statement is too broad to add value to the paper. Without a clear definition of illumination and quality, this path for justification will always suggest that image software is lacking.

***Reply:*** *We have revised this description in L93-99.*

11. The authors suggest that the method in Warman et al. [27] is not suitable for field applications? Does this refer to the software? The imaging platform? Again, the authors are looking to justify their software, they need to be more specific.
***Reply:*** *Recently, the use of deep learning methods has achieved good results [27], but this approach is limited by the simplicity of the imaging platform (a non-product concept device) and low analysis efficiency (one ear per analysis). We addressed this in L101-103.*

12. Consider one of the following: marginalize the software and imaging components of the paper which will reduce their need for justification or spend the time to highlight the novel aspects of MAIZTRO which are not available elsewhere and are important to the discoveries in the paper. For example: a) MAIZTRO produces a kernel count where [26] does not b) MAIZTRO highlights different regions of the ear where others [26] do not. Highlighting where the community needs help is a fine way to justify the creation of novel software. I encourage the authors to be more specific in this part of the paper.

***Reply:*** *In the Results and Discussion sections, we have modified the description of various aspects of MAIZTRO and outlined its advantages over other tools. We have added the training method and data allocation ratio for the model at L129-130. We have added shooting methods at L136-L137 (photos were taken of both the front and back of each ear). We have added a flowchart to describe the relationship between different layers of MAIZTRO and provided information about the operating system and programming language at L138-139 and Fig. S13.* *We also added information about how MAIZTRO prevents overfitting at L129-130, L144-148 and L394-432. We summarized the advantages of MAIZTRO in L87-103 and L276-287.*

13. The authors suggest that an integrated software and hardware platform is needed. Why? While this may seem trivial at first thought, careful consideration reveals integration between the data acquisition and analysis should be carefully considered. If integration is deemed the best approach, then providing the readers with an understanding of how the data acquisition software integrates with the downstream analysis software is important. For example: Did the authors choose a “modular” integration between these layers so that problems in the image analysis would not affect the data acquisition? Can the analysis layer be shared with the public or is the software system not modular in nature? Other simple details are missing from the text, such as: What language is the analysis written in? What operating system is the system running? What resolution are the images?

***Reply:*** *We emphasize the integration of software and hardware to show that MAIZTRO has most of the steps needed for complete ear analysis: sample information input, sample placement, image scanning, image analysis, and downloaded data. This integration is very important for MAIZTRO to be used in practical work and handle the phenotypic analysis of maize ears robustly. In addition, integration is convenient for operation and training, which allows users to start the measurement of ear phenotypes after simple training without prior complex knowledge. To illustrate how the data acquisition software integrates with the downstream analysis software, we have added a flow chart in the Supplementary Information (Fig. S13).*

**Fig. S13 Flow chart of relationship between different layers of MAIZTRO.**

*Data acquisition and image analysis are modularized and integrated separately. An interactive software interface is used to operate the three layers to realize data transmission and transformation between the three layers. The operating software first controls the camera to scan the ears and obtain images. It then loads the analysis model to analyze the images and finally exports the data as a dataset through the operating software. Each operation has a corresponding button. Therefore, if there is a problem in the analysis layer, we can still use the interactive interface to obtain the image of an ear and save it. When the analysis algorithm is reconfigured, we can continue the analysis process. In addition, we can improve the analysis* *algorithm to further improve accuracy and efficiency without adjusting all the parts.*

*Our software system is designed modularly to integrate the functions of each part. Our software and analysis algorithms are written in Python and run on a Windows platform. Each ear image is 3408 × 2048 pixels. At present, we have not considered open-source software and analysis code.*

14. The paper suggests that "in field" imaging is lacking however the pictures of the imaging device are shown as being used indoors. Please specify what "in field" means, why it is important, why they believe it is necessary and how it is different from "out of field".

***Reply:*** *Breeding is usually done on a large scale in the field, whether farmland or an experimental station. Ear testing is not usually done in a lab, as this can involve transportation problems and introduce uncertainty from problems like damage to the ears. By "in field" we mean testing is done on-site at a farm or experimental station, although the MAIZTRO would generally be set up in a simple room nearby, largely to provide a power supply and protection from weather (such as rainfall). In contrast, "out of field" testing refers to work done in the laboratory, where conditions are far more controlled than for in-field (on-site) testing.*

*Most breeding efforts involve growing large quantities of breeding material, which is traditionally analyzed by the breeder or with the help of field staff. Because finding experienced field staff can be difficult, data records often lack detail and are subjective. MAIZTRO is developed to address these problems by allowing breeders and their staff to measure ear characteristics of any material comprehensively, without subjectivity and sampling errors, due to its mobility, ease of use, efficiency, and the comprehensiveness of its indicators.*

15. The authors present a table with metrics for the algorithms however it is unclear with respect to the training, testing or validation. What percent of the data was held out during training? Was attention given to performing structured hold-outs by genotype or variety? For example, what happens if the authors leave out the tropical variety and only train on the rest? Do their metrics change? What if they leave out dent corn? Overall, it is the authors responsibility to demonstrate they have not over fit their models. Currently, the paper is lacking in this respect. Please provide more information to ensure the reader that the model is not overfitting the data.
***Reply:*** *During training of the model, we distribute the data in a ratio of 8:2 (training and validation) and use cross-validation to train the parameters of the model, which can offer a more stable model over time (Müller and Guido, 2016). The model precision table shown in the first part of our results (Table 1) comes from a new data set that did not appear in the training. After the model parameters are trained based on cross-validation, they are used in the dataset for validation. It should be noted, therefore, that the precision table actually reflects how well the model can be generalized. In calculating accuracy, we refer to the recommendations of Kalantar-Zadeh [28] and use multiple parameters to reflect the accuracy and stability of* *MAIZTRO, considering the effects of different operators, different MAIZTRO devices, measurement duration, and the environment on accuracy. Refer to the Methods section for detailed calculations and measurements and the first section of the Results (P6).* *In addition, we also analyzed the performance of MAIZTRO on different types of ears in the fifth question of reviewer 1.*

16. The data on github seems well organized however understanding the function of different scripts, what the structure of the data is and how to interact with the data is missing. Please fill out the readme file. This should include detailed information about how to use the data. A well documented large dataset alone has value for the community and will enable if if time is taken to document how to load the data.

*Reply: We have added a README.md file to the packages in github, which can be found through the link we provide in the section on Availability of data and materials.*

17. I repeat, few of the above suggestions change the conclusions of the paper: high-throughput image measurement of transgenic corn ears uncovered genes potentially involved in yield plasticity. The results presented here are important however details and justification could use attention.
Below is a list of necessary fixes:
a: Ensure the reader that the extraction method is not overfitting the data. Provide structured holdouts by randomizing varieties and holding out varieties.
b: Include more details, for example the resolution of the images.
c: Provide a better understanding of the term "in-field".

***Reply:*** *Thank you for this summation. We have answered these questions in detail as follows:*

a) *Data are distributed 8:2 during model training, with cross-validation applied when training the model parameters, so that a more stable model can be achieved (Müller and Guido, 2016). The model precision table (Table 1) in the first part of our results comes from a new data set that hasn't appeared in the training. After cross-validation, model parameters are used in the dataset for validation. Thus, the precision table actually reflects the model's generalizability. In calculating the accuracy, we refer to the recommendations of Kalantar-Zadeh [28] and use multiple parameters to reflect the accuracy and stability of MAIZTRO, taking into account the effects of different operators, different MAIZTRO devices, measurement duration, and the environment. For detailed calculations and measurements, refer to the Methods* *section. In addition, we also analyzed the performance of MAIZTRO on different types of ears in the fifth question of reviewer 1.*
b) *We have added this information in the revised manuscript and added a flow chart to analyze and display the relationship between different layers of MAIZTRO (Fig. S13) and the* *software and operating system information in the first section of the Results (P6-7, Fig. S13).*
c) *We address this in detail in our response to question 14 above.*

18. Please remove the word "urgent" on line 97.

***Reply:*** *We have removed this word.*

***References:***

*Avila Bolivar, L M. (2016). Molecular Analyses of Internode Elongation and Cold Stress Tolerance in Maize. University of Guelph.*

*Avila, L., Obeidat, W., Earl, H., Niu, X., Hargreaves, W., & Lukens, L. (2018). Shared and genetically distinct Zea mays transcriptome responses to ongoing and past low temperature exposure. BMC Genomics, 19(1). doi:* [*https://doi.org/10.1186/s12864-018-5134-7*](https://doi.org/10.1186/s12864-018-5134-7)

*Crossa, J. (1990). Statistical analyses of multilocation trials. Advances in Agronomy 44, 55-85. doi: https://doi.org/10.1016/s0065-2113(08)60818-4*

*Du, Q., Wang, K., Xu, C., Zou, C., Xie, C., Xu, Y., & Li, W. (2016). Strand-specific RNA-Seq transcriptome analysis of genotypes with and without low-phosphorus tolerance provides novel insights into phosphorus-use efficiency in maize. BMC Plant Biology, 16(1). doi: https://doi.org/10.1186/s12870-016-0903-4*

*Frey, F. (2017). The genetic basis of heat tolerance in temperate maize (Zea mays L.). Retrieved 23 October 2022, from* [*http://opus.uni-hohenheim.de/volltexte/2017/1299/*](http://opus.uni-hohenheim.de/volltexte/2017/1299/)

*Gage, J., Jarquin, D., Romay, C., Lorenz, A., Buckler, E., & Kaeppler, S. et al. (2017). The effect of artificial selection on phenotypic plasticity in maize. Nature Communications, 8(1). doi: https://doi.org/10.1038/s41467-017-01450-2*

*Huang C., Sun H., Xu D., Chen Q., Liang Y., Wang X. et al. (2018). ZmCCT9 enhances maize adaptation to higher latitudes. Proceedings of the National Academy of Sciences, 115(2), E334-E341. doi:* [*https://doi.org/10.1073/pnas.171805811*](https://doi.org/10.1073/pnas.171805811)

*Hufford, M., Xu, X., van Heerwaarden, J., Pyhäjärvi, T., Chia, J., & Cartwright, R. et al. (2012). Comparative population genomics of maize domestication and improvement. Nature Genetics, 44(7), 808-811. doi: https://doi.org/10.1038/ng.2309*

*Jia, Z., Yang, X., Hou, X., Nie, Y., & Wu, J. (2022). The importance of a genome-wide association analysis in the study of alternative splicing mutations in plants with a special focus on maize. International Journal of Molecular Sciences, 23(8), 4201.doi:* [*https://doi.org/10.3390/ijms23084201*](https://doi.org/10.3390/ijms23084201)

*Kusmec, A., Srinivasan, S., Nettleton, D., & Schnable, P. (2017). Distinct genetic architectures for phenotype means and plasticities in Zea mays. Nature Plants, 3(9), 715-723. doi: https://doi.org/10.1038/s41477-017-0007-7*

*Liu, N., Du, Y., Warburton, M., Xiao, Y., & Yan, J. (2020). Phenotypic plasticity contributes to maize adaptation and heterosis. Molecular Biology and Evolution,38(4), 1262-1275. doi: https://doi.org/10.1093/molbev/msaa283*

*Ma, L., Zhang, M., Chen, J., Qing, C., He, S., & Zou, C. et al. (2021). GWAS and WGCNA uncover hub genes controlling salt tolerance in maize (Zea mays L.) seedlings. Theoretical and Applied Genetics, 134(10), 3305-3318. doi: https://doi.org/10.1007/s00122-021-03897-w*

*Müller A C, Guido S. (2016). Introduction to Machine Learning with Python: A Guide for Data Scientists[M]. " O'Reilly Media, Inc.".*

*Makanza, R., Zaman-Allah, M., Cairns, J., Eyre, J., Burgueño, J., & Pacheco, Á. et al. (2018). High-throughput method for ear phenotyping and kernel weight estimation in maize using ear digital imaging. Plant Methods, 14(1). doi: https://doi.org/10.1186/s13007-018-0317-4*

*Miller, N.D., Haase, N.J., Lee, J., Kaeppler, S.M., de Leon, N., Spalding, E.P. (2017). A robust, high-throughput method for computing maize ear, cob, and kernel attributes automatically from images. The Plant Journal, 89,169–178. doi: https://doi.org/10.1111/tpj.13320*

*Song, K., Kim, H., Shin, S., Kim, K., Moon, J., Kim, J., & Lee, B. (2017). Transcriptome analysis of flowering time genes under drought stress in maize leaves. Frontiers in Plant Science, 8. doi:* [*https://doi.org/10.3389/fpls.2017.00267*](https://doi.org/10.3389/fpls.2017.00267)

*Sun G, Wase N, Shu S, et al. (2021). The genome of stress tolerant crop wild relative Paspalum vaginatum leads to increased biomass productivity in the crop Zea mays[J]. bioRxiv.*

*Wang, W., Lu, Y., Li, J., Zhang, X., Hu, F., Zhao, Y., & Zhou, D. X. (2021). SnRK1 stimulates the histone H3K27me3 demethylase JMJ705 to regulate a transcriptional switch to control energy homeostasis. The Plant Cell, 33(12), 3721-3742. doi:* [*https://doi.org/10.1093/plcell/koab224*](https://doi.org/10.1093/plcell/koab224)

*Warmm, C., Sullivan C.M., Preece J., Buchanan M. E., Vejlupkova, Z., Jaiswal, P., Flowler, J.E. (2021). A cost-effective maize ear phenotyping platform enables rapid categorization and quantification of kernels. The Plant Journal, 106, 566-579. doi:* [*https://doi.org/10.1111/tpj.15166*](https://doi.org/10.1111/tpj.15166)

*Wei, K., Wang, Y., & Xie, D. (2013). Identification and expression profile analysis of the protein kinase gene superfamily in maize development. Molecular Breeding, 33(1), 155-172. doi:* [*https://doi.org/10.1007/s11032-013-9941-x*](https://doi.org/10.1007/s11032-013-9941-x)

*Wei, L., Zhang, X., Zhang, Z., Liu, H., & Lin, Z. (2018). A new allele of the Brachytic2 gene in maize can efficiently modify plant architecture. Heredity, 121(1), 75-86. doi: https://doi.org/10.1038/s41437-018-0056-3*

*Yang, Q., Li, Z., Li, W., Ku, L., Wang, C., Ye, J., et al. (2013). CACTA-like transposable element in ZmCCT attenuated photoperiod sensitivity and accelerated the postdomestication spread of maize. Proceedings of the National Academy of Sciences, 2013, 110(42): 16969-16974. doi:* [*https://doi.org/10.1073/pnas.1310949110*](https://doi.org/10.1073/pnas.1310949110)

*Zhang, Z., Zhang, X., Lin, Z., Wang, J., Xu, M., Lai, J., Yu, J., & Lin, Z. (2018). The genetic architecture of nodal root number in maize. The Plant Journal, 93(6), 1032–1044.* [*https://doi.org/10.1111/tpj.13828*](https://doi.org/10.1111/tpj.13828)*.*

**Second round of review**

**Reviewer 1**

I do not have more questions for the authors. The authors answered most of my questions and comments except the phenotypes based on gene editing materials. The authors said that it will take years to get these materials. While this is true, but I still suggest that the authors go futher for this part in their future studies even after the publication of this paper, because the convincing proofs from the knockout materials will make the conclusion stronger.

**Reviewer 2**

The manuscript moves forward the area of ear phenotyping and plasticity. It could benefit the community and the paper if the images in the manuscript were made public.

Below are a few more suggestions which pick at the justification for a new system. In brief, Miller et al does not measure total kernels per ear or kernel row number, Tanabata et al was not made for kernels on the ear, and Warman et al was not made for high-throughput. Therefore,, the authors see a need for a system that meets two criteria: 1) The ability to image more than 10,000 ears per day 2) Measure ear traits outlined in Miller et al and kernel counts outlined in Warman et al combined in one system.

In expanding on this simple statement, the authors are introducing erroneous statements about each of the above. Some of these errors are listed below. Perhaps a simple statement without details would avoid the below tangled statements.

[L95-97]: However, using such methods based on gray signal periodicity to analyze kernel characteristics is limited by the object's color and distribution.

Two parts of this sentence are still mixed:
Are the author’s suggesting the fourier analysis is limited by the color of the object? If so, please specify how.
The authors suggest the distribution is limiting analysis? If so, the distribution of what and how does it limit?

[L94-95]: …with machine learning technology (e.g., using Fast Fourier Transform).

The FFT method is not typically considered among machine learning methods. Are the authors suggesting Miller et al “learned” fourier coefficients or combined a deterministic FFT method with a learning method to analyze the ear?

[L97-99]: The calculation of kernel numbers mainly depends on the counting of separate kernels, which inevitably increases the workload. In addition, the configuration of the system is relatively complex and has relatively high user requirements.

From my understanding, Miller et al does not count the total number of kernels on the ear. While the kernel method discussed here does take time, it appears to be used for extracting kernel based features which are not accessible when the kernels are on the ear. That the author’s method accurately counts total kernel number is an advancement over Miller et al. Miller et al, does count kernels per row but does not count the kernel row number.
It is unclear what the authors mean by “relatively complex” and “ relatively high user requirements”.

[L188-189] There were strong positive correlations among ear diameter, ear width, and 189 ear circumference….
Given that the ear is locally cylindrical, these are expected correlations and are not noteworthy. These observations are equivalent to saying that the circumference of a circle is related to the radius or diameter. In a sense these are not biological correlations but rather mathematical in nature.

**Authors Response**

**Point-by-point responses to the reviewers’ comments:**

Reviewer 1
I do not have more questions for the authors. The authors answered most of my questions and comments except the phenotypes based on gene editing materials. The authors said that it will take years to get these materials. While this is true, but I still suggest that the authors go futher for this part in their future studies even after the publication of this paper, because the convincing proofs from the knockout materials will make the conclusion stronger.
*Reply: Thanks for your suggestion. We agree with your suggestion, which will be very helpful when studying functional genes. We will continue to study these genes over the next 3-4 years and work with our team to perform detailed gene verification and gene function analysis. It's hoped that we'll be able to submit our latest findings about detailed gene function analysis to the journal in the future.*

Reviewer 2
The manuscript moves forward the area of ear phenotyping and plasticity. It could benefit the community and the paper if the images in the manuscript were made public. Below are a few more suggestions which pick at the justification for a new system. In brief, Miller et al does not measure total kernels per ear or kernel row number, Tanabata et al was not made for kernels on the ear, and Warman et al was not made for high-throughput. Therefore,, the authors see a need for a system that meets two criteria: 1) The ability to image more than 10,000 ears per day 2) Measure ear traits outlined in Miller et al and kernel counts outlined in Warman et al combined in one system.

In expanding on this simple statement, the authors are introducing erroneous statements about each of the above. Some of these errors are listed below. Perhaps a simple statement without details would avoid the below tangled statements.

[L95-97]: However, using such methods based on gray signal periodicity to analyze kernel characteristics is limited by the object's color and distribution.
Two parts of this sentence are still mixed:
Are the author’s suggesting the fourier analysis is limited by the color of the object? If so, please specify how.
The authors suggest the distribution is limiting analysis? If so, the distribution of what and how does it limit?

[L94-95]: …with machine learning technology (e.g., using Fast Fourier Transform).

The FFT method is not typically considered among machine learning methods. Are the authors suggesting Miller et al “learned” fourier coefficients or combined a deterministic FFT method with a learning method to analyze the ear?

[L97-99]: The calculation of kernel numbers mainly depends on the counting of separate kernels, which inevitably increases the workload. In addition, the configuration of the system is relatively complex and has relatively high user requirements.

From my understanding, Miller et al does not count the total number of kernels on the ear. While the kernel method discussed here does take time, it appears to be used for extracting kernel based features which are not accessible when the kernels are on the ear. That the author’s method accurately counts total kernel number is an advancement over Miller et al. Miller et al, does count kernels per row but does not count the kernel row number.
It is unclear what the authors mean by “relatively complex” and “ relatively high user requirements”.
*Reply:* *The reviewer's concerns are about the fifth paragraph of the background section, which are related to the analysis of the new system's justification. We appreciate the detailed comments made by this reviewer, who points out some tangled statements in our summary, refines and summarizes them, and makes some insightful suggestions for revision. The mentioned part in the paragraph has been revised following the suggestions of the reviewers.
The updated statements are summarized as follows (L87-L106 in the revised version): Although some previous studies have tried to explore the effectiveness of automated measurement methods, they are limited by efficiency or lack of several key ear phenotypes. One such method treats images on a homogenous background with traditional image processing technology, capturing each kernel as an object and obtaining the length and width of the kernel [26]. It can be difficult to use this method to obtain maize kernel objects on an ear, however, due the nonhomologous background (i.e., other kernels on the ear). Miller et al. [27] have solved this problem; however, they ignored key ear traits such as the number of kernels per ear and the number of rows per ear. Warman et al. [28] proposed novel algorithms to focus on these ear traits, but the approach is limited by the simplicity of the imaging platform (a non-product concept device) and low analysis efficiency (one ear per analysis). Therefore, there is a need to develop a high-throughput automated phenotyping platform that integrates the ear phenotypes concerned by Miller et al. [27] and Warman et al. [28], as well as many other critical phenotypic characteristics.*

[L188-189] There were strong positive correlations among ear diameter, ear width, and 189 ear circumference
Given that the ear is locally cylindrical, these are expected correlations and are not noteworthy. These observations are equivalent to saying that the circumference of a circle is related to the radius or diameter. In a sense these are not biological correlations but rather mathematical in nature.
*Reply: Yes, we have deleted this sentence.*
*Please accept our gratitude for the efforts you have made to improve this article.*

**Third round of review**

**Reviewer 2**

The authors have addressed the concerns.
